# Correction to: De novo pyrimidine synthesis is a collateral metabolic vulnerability in *NF2*-deficient mesothelioma

**DOI:** 10.1038/s44321-025-00366-5

**Published:** 2026-01-16

**Authors:** Duo Xu, Yanyun Gao, Shengchen Liu, Shiyuan Yin, Tong Hu, Haibin Deng, Tuo Zhang, Balazs Hegedüs, Thomas M Marti, Patrick Dorn, Shun-Qing Liang, Ralph A Schmid, Ren-Wang Peng, Yongqian Shu

**Affiliations:** 1 Department of Oncology, The First Affiliated Hospital of Nanjing Medical University, Nanjing, China; 2 Department of General Thoracic Surgery, Inselspital, Bern University Hospital, University of Bern, Bern, Switzerland; 3 Department for BioMedical Research (DBMR), Inselspital, Bern University Hospital, University of Bern, Bern, Switzerland; 4 Department of Cardiothoracic Surgery, Nanjing First Hospital, Nanjing Medical University, Nanjing, China; 5 Department of Thoracic Surgery, University Medicine Essen - Ruhrlandklinik, West German Cancer Center, University Hospital Essen, University Duisburg-Essen, Tüschener Weg 40, 45239 Essen, Germany; 6 Department of Medicine, University of Minnesota Twin Cities, 516 Delaware Street SE, Minneapolis, MN USA

## Abstract

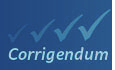

**Correction to:** *EMBO Molecular Medicine* (2025) 17:2258–2298. 10.1038/s44321-025-00278-4 | Published online 24 July 2025

The authors notified the journal after finding an error in one of the figure panels.

After reviewing the supplied source data the journal agrees to withdraw and replace Fig. EV1C.

**Figure EV1C is withdrawn and replaced**.

**Figure EV1C:**
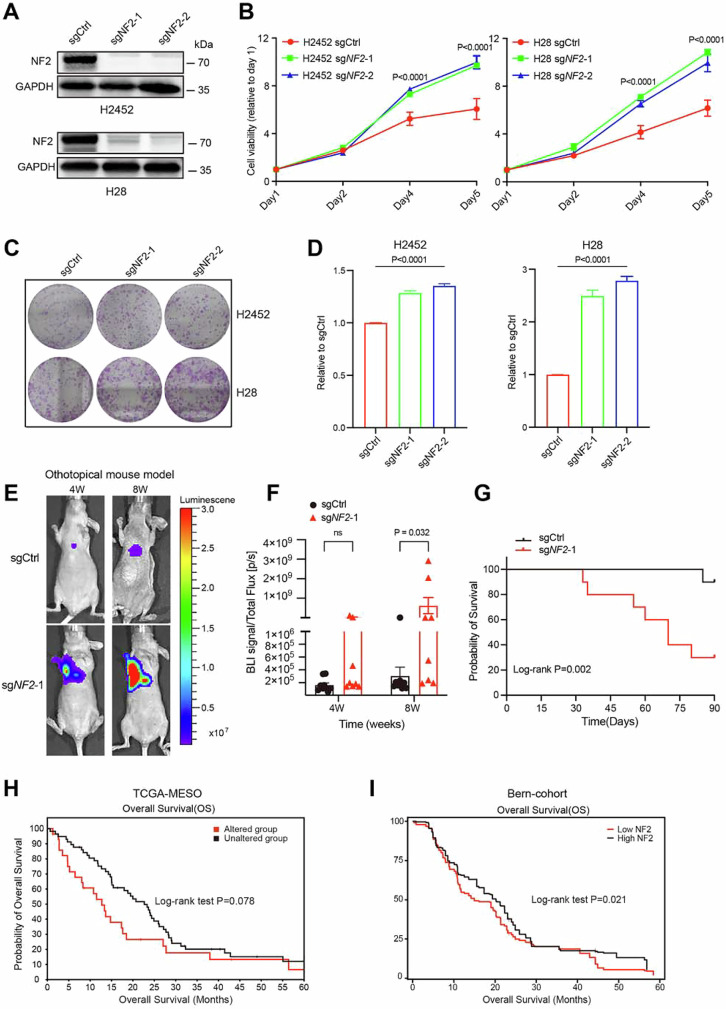
**Original published image**. Source data are available online for this figure.

**Figure EV1C:**
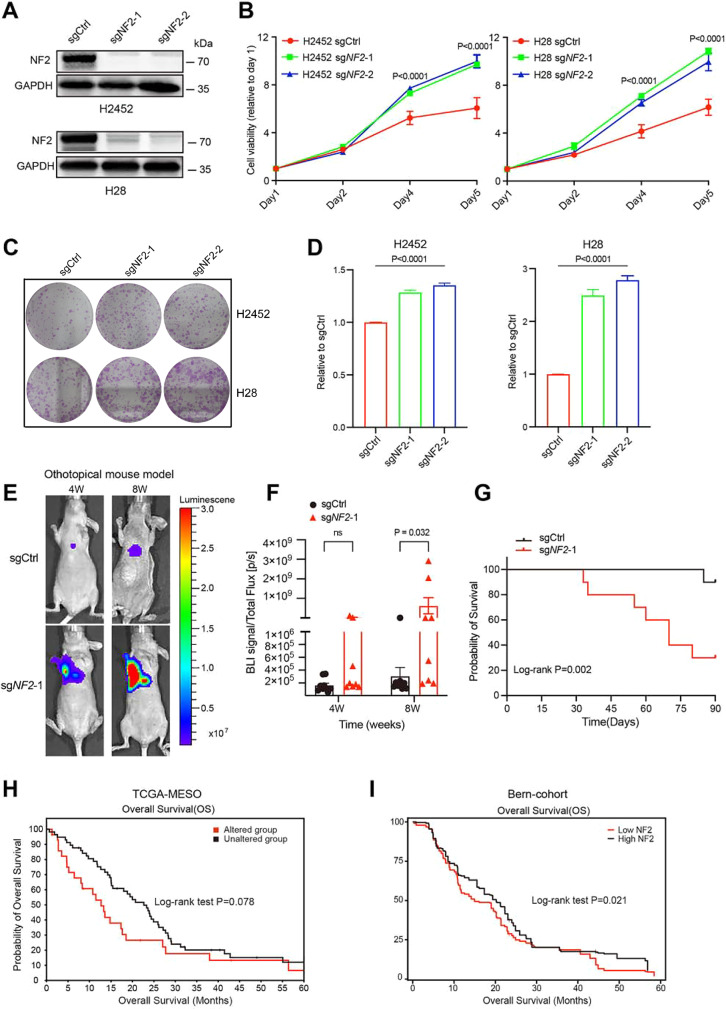
**Corrected**. Source data are available online for this figure.

**Corresponding EV1C source data is published with this Author Correction.**

**Author statement:**

During a post-publication review, the authors discovered that one panel in Fig. EV1C (*H2452, sgCtrl*) was inadvertently duplicated from Fig. 3D (*H2452, sgCtrl_DHODHi*) during the figure assembly process. The image shown in Fig. 3D is correct, while the panel in Fig. EV1C was mistakenly inserted.

The authors have verified the original raw data and prepared a corrected version of Fig. EV1C.

This correction does not affect the results, interpretation, or conclusions of the study.

The authors apologize for these errors.

All authors agree with this corrigendum.

## Supplementary information

Figure EV1C Source Data

