# Supplementary material for: De novo pyrimidine synthesis is a collateral metabolic vulnerability in NF2-deficient mesothelioma
Source: EMBO Mol Med. 2025 Jul 24;17(9):2258–98. doi: 10.1038/s44321-025-00278-4 (PMC12423300; doi:10.1038/s44321-025-00278-4)
Supplement: Supplementary file 1 — Appendix [file 44321_2025_278_MOESM1_ESM.pdf]

Appendix for "*De Novo* Pyrimidine Synthesis Is a Collateral Metabolic Vulnerability in *NF2*-deficient Mesothelioma"

Table of Contents

1. **Appendix Figure S1..Page 2**
2. **Appendix Figure S2..Page 3**
3. **Appendix Figure S3..Page 4-5**
4. **Appendix Figure S4..Page 6-7**
5. **Appendix Figure S5..Page 8-9**
6. **Appendix Figure S6..Page 10**
7. **Appendix Figure S7..Page 11**

Appendix Figure S1

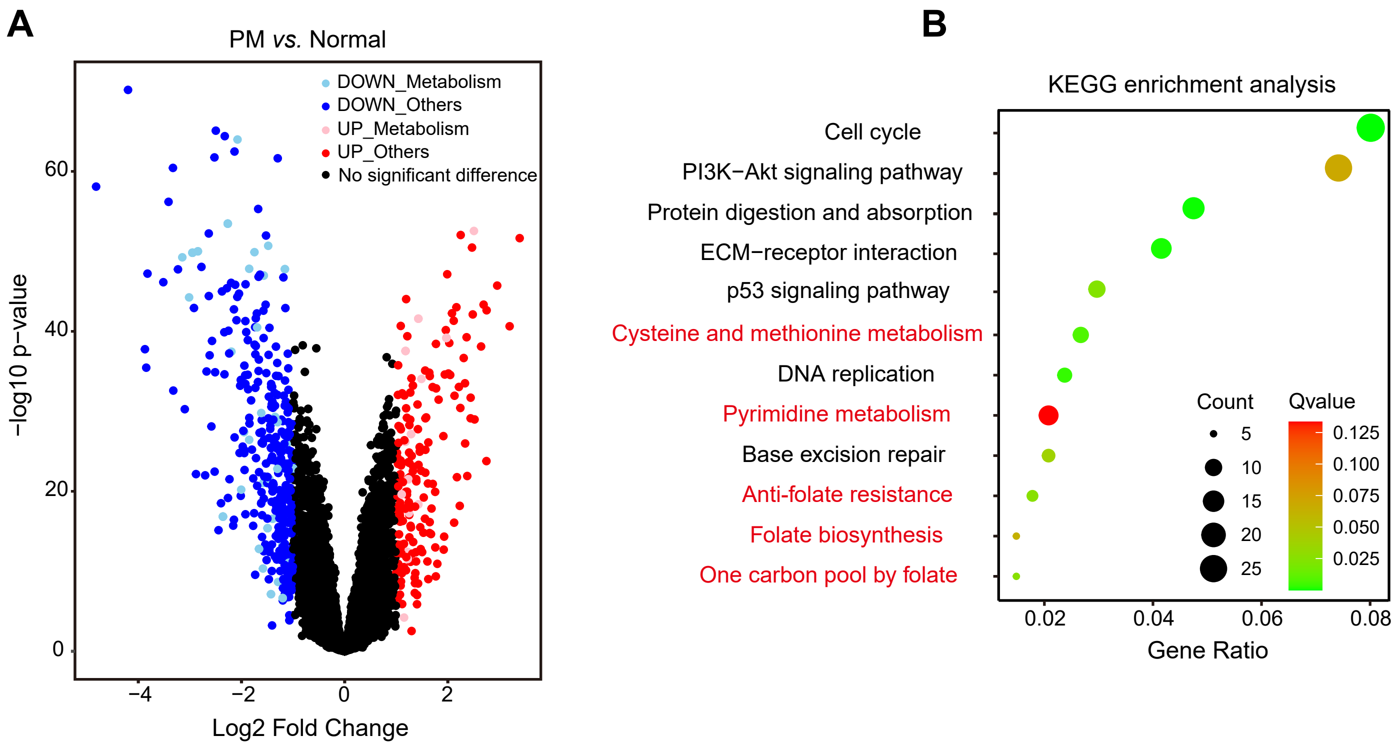

Appendix Figure S1: Molecular alterations in pleural mesothelioma tumors.

(A) Volcano plot showing the genes downregulated (adjusted p-value (padj) <0.05 and log2-fold change<-1) (in blue; metabolic-related in light blue) and upregulated (padj<0.05 and log2-fold change>1) (in red; metabolic-related in light red) in PM tumors (n=328) compared to normal tissues (n=65; pleura: 22; lung:43). Transcriptomic data from pleural mesothelioma (PM) patients with high-coverage gene profiles (gene number>20,000) were obtained from The Cancer Genome Atlas (TCGA) MESO dataset, the Gene Expression Omnibus (GEO; GSE12345, GSE163720, GSE42977, and GSE51024) and the European Bioinformatics Institute (EMBL-EBI; E-MTAB-1109).

(B) Kyoto Encyclopedia of Genes and Genomes (KEGG) enrichment analysis was conducted on differentially expressed genes (DEGs) (padj<0.05 and log2-fold change<-1 or>1) in PM tumors (n=328) compared to normal tissues. Metabolic-related pathways are marked in red. The gene ratio represents the number of DEGs relative to the total number of annotated genes within the specified pathway.

Appendix Figure S2

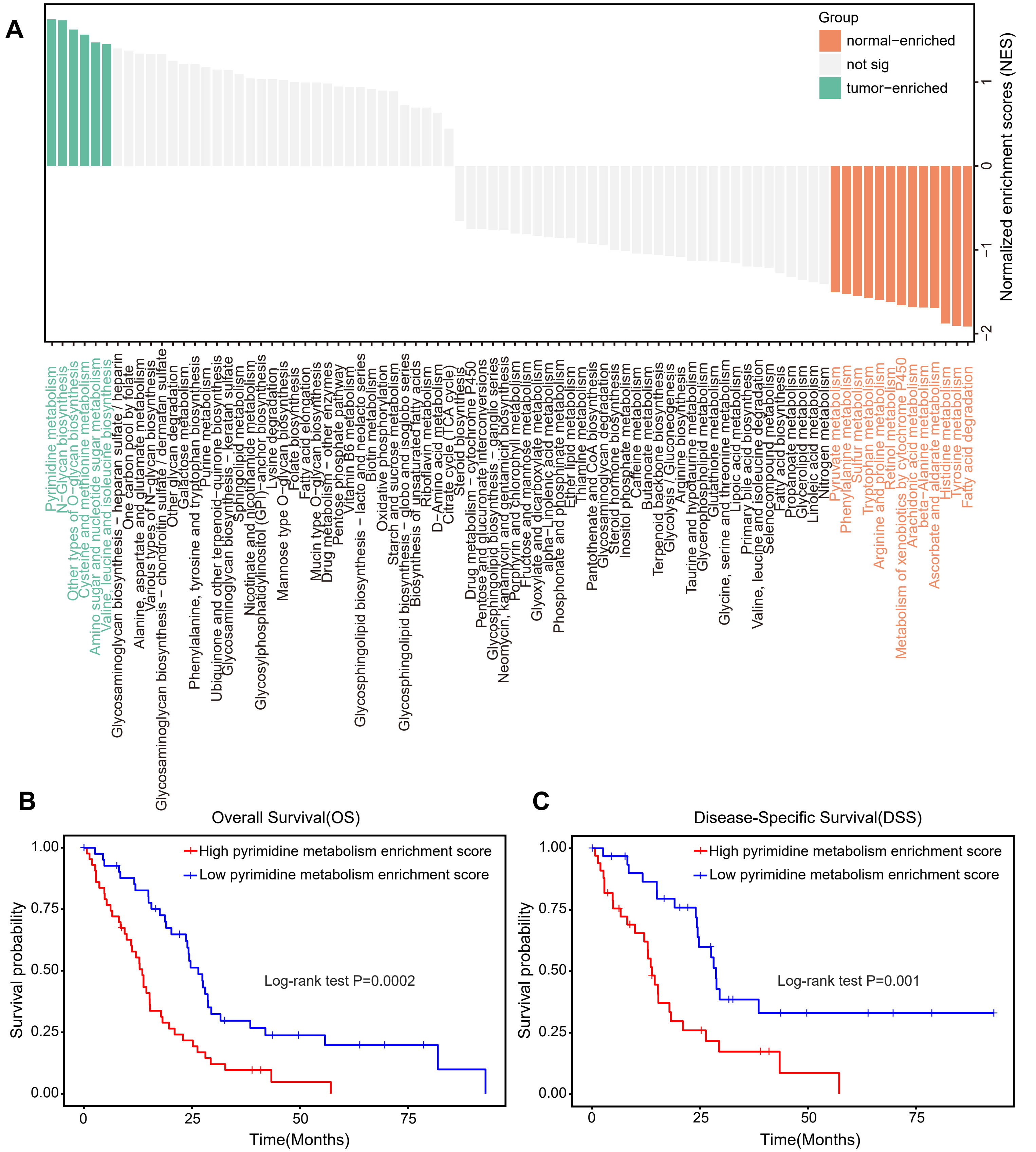

Appendix Figure S2. Metabolic reprogramming in PM tumors.

(A) Bar plots showing the differential normalized enrichment scores of 84 metabolic pathways in PM tumors (n=3 compared to normal tissues (n=65). The significantly upregulated (in green) and downregulated metabolic pathways (in orange) with padj<0.05 are marked.

(B,C) Kaplan–Meier univariate overall survival (B) and disease-specific survival (C) analyses among PM patients. The median values of enrichment scores across all patients were used to stratify the PM cohort. Clinical data were obtained from the TCGA-MESO cohort (n=87). The p-value was calculated by the log-rank test in R.

Appendix Figure S3

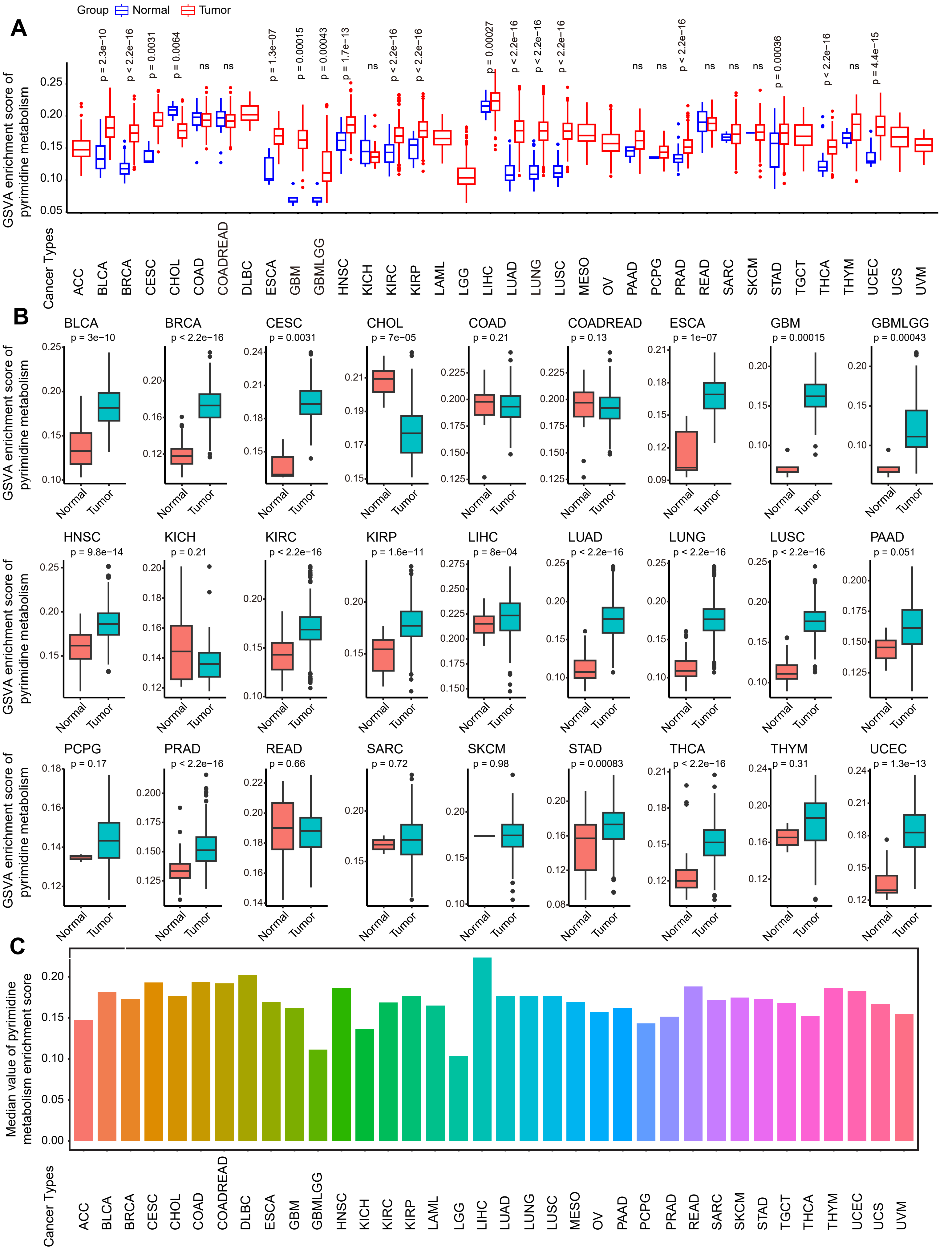

Appendix Figure S3. Dysregulated pyrimidine biosynthesis pathway in the pan-cancer cohort.

(A, B) Box plots showing the differences in the gene set variation analysis (GSVA) enrichment scores for pyrimidine metabolism between normal and tumor samples in the pan-cancer cohort (n=36, shown in A). The comparisons of independent cancer types with normal samples are shown separately in (B). The Wilcoxon rank-sum test was used for comparison. ns: not significant.

(C) Bar plots showing the median values of pyrimidine metabolism enrichment scores in tumor samples across the pan-cancer cohort (n=36). ACC: adrenocortical carcinoma; BLCA: bladder urothelial carcinoma; BRCA: breast invasive carcinoma; CESC: cervical squamous cell carcinoma and endocervical adenocarcinoma; CHOL: cholangiocarcinoma; COAD: colon adenocarcinoma; DLBC: lymphoid neoplasm diffuse large B-cell lymphoma; ESCA: esophageal carcinoma; GBM: glioblastoma multiforme; HNSC: head and neck squamous cell carcinoma; KICH: kidney chromophobe; KIRC: kidney papillary cell carcinoma; KIRP: kidney renal papillary cell carcinoma; LAML: acute myeloid leukemia; LGG: brain lower grade glioma; LIHC: liver hepatocellular carcinoma; LUAD: lung adenocarcinoma; LUSC: lung squamous cell carcinoma; MESO: mesothelioma; OV: ovarian serous cystadenocarcinoma; PAAD: pancreatic adenocarcinoma; PCPG: pheochromocytoma and paraganglioma; PRAD: prostate adenocarcinoma; READ: rectum adenocarcinoma; SARC: sarcoma; SKCM: skin cutaneous melanoma; STAD: stomach adenocarcinoma; TGCT: testicular germ cell tumor; THCA: thyroid carcinoma; THYM: thymoma; UCEC: uterine corpus endometrial carcinoma; UCS: uterine carcinosarcoma; UVM: uveal melanoma.

Appendix Figure S4

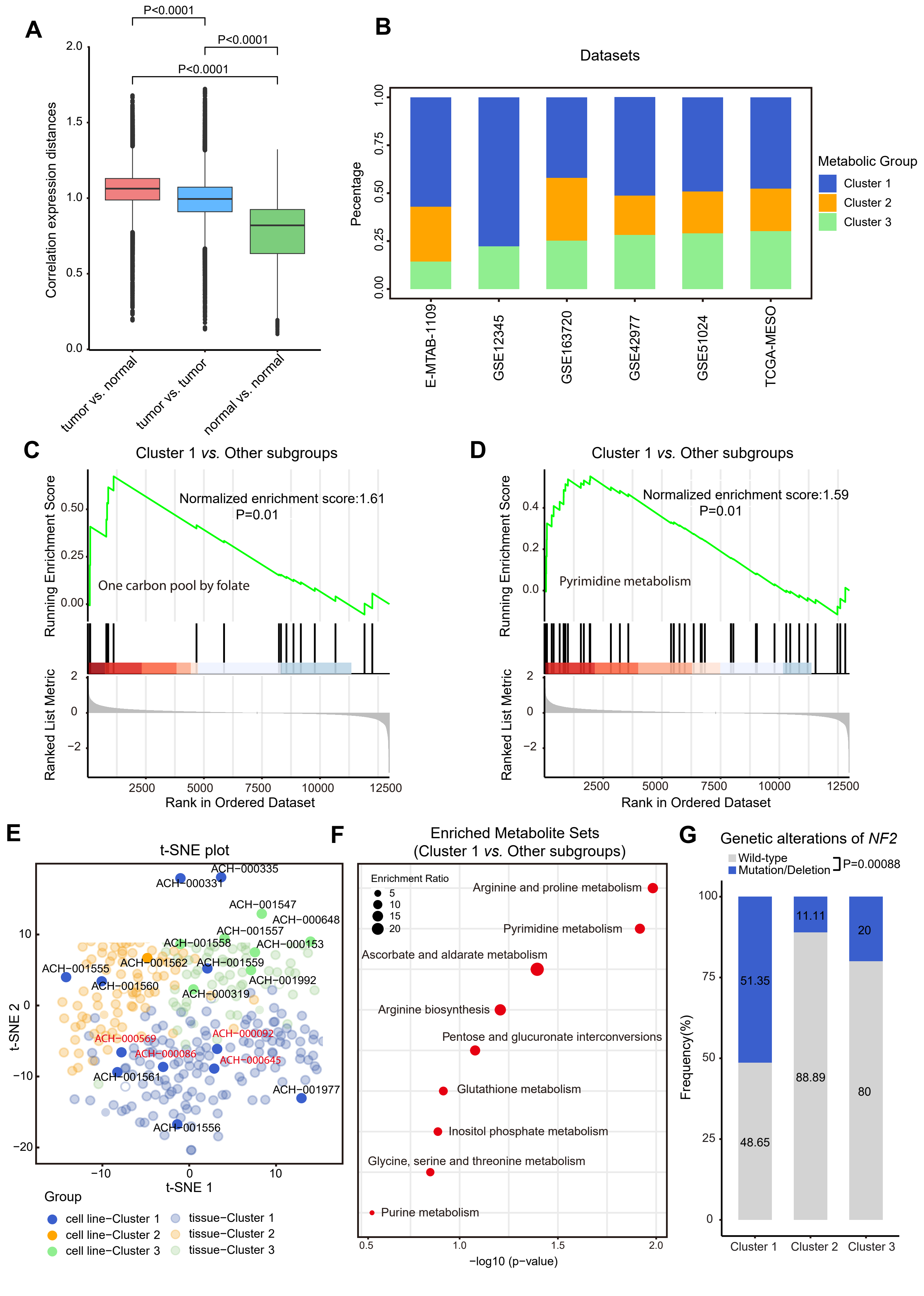

Appendix Figure S4. Metabolic heterogeneity in PM tumors.

(A) Euclidean expression distances of metabolic genes between PM tumors (n=328) and normal tissues (n=65). One-way ANOVA with multiple comparisons was used for statistical analysis.

(B) Bar plots showing the distribution of metabolic subgroups within different PM datasets. Transcriptomic data from PM patients with high-coverage gene profiles (more than 20,000 genes) were obtained from The Cancer Genome Atlas (TCGA) MESO dataset, the Gene Expression Omnibus (GEO; GSE12345, GSE163720, GSE42977, and GSE51024) and the European Bioinformatics Institute (EMBL-EBI; E-MTAB-1109).

(C,D) Representative gene set enrichment analysis (GSEA) plots showing a significant upregulation of one carbon pool by folate (C) and pyrimidine metabolism (D) in PM tumors classified as Cluster 1 compared to those in Clusters 2 and 3 metabolic subtypes.

(E) t-Distributed stochastic neighbour embedding (t-SNE) plot showing the metabolic pathway-based clustering in PM cell lines (n=19) and PM tumor tissues(n=328). PM cell lines that accurately categorized into Cluster 1 and are linked to the publicly accessible metabolite dataset are marked in red (n=4). The other three cell lines that also belong to Cluster 1 but lack a metabolite dataset were omitted from the following analyses.

(F) KEGG enrichment analysis of differentially expressed metabolites with a p- value<0.2 and a fold change>0.25 in PM cell lines classified as Cluster 1 with the publicly available metabolite dataset ((n=4; highlighted in (E)) compared to cell lines from other metabolic subtypes.

(G) The percentage of *NF2* genetic alterations (mutations/deletions) across different metabolic subgroups was analyzed using a hypergeometric test for comparison analysis.

Appendix Figure S5

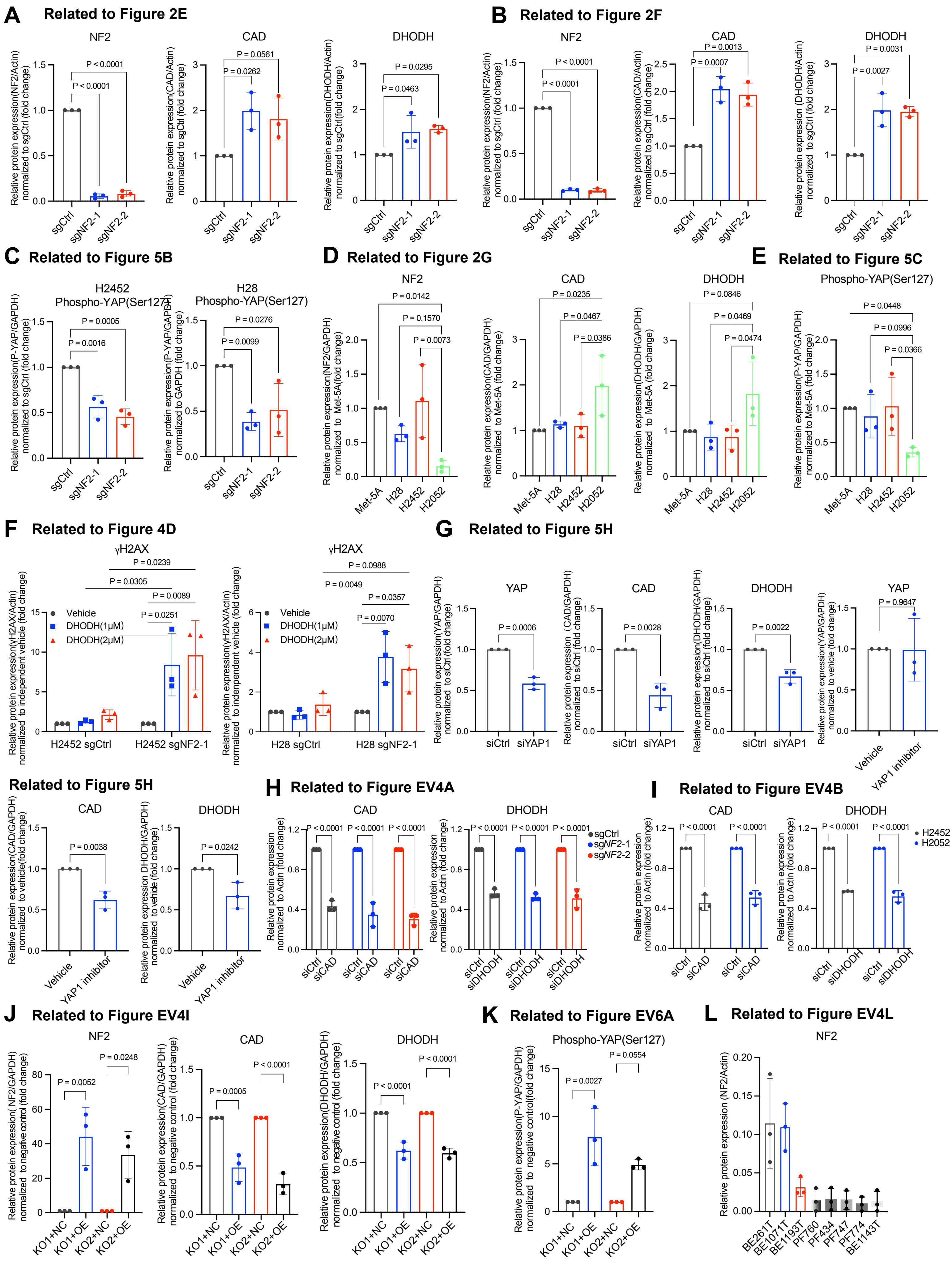

Appendix Figure S5. Quantification of the presented Western blots.

(A-C) Quantification data from immunoblots of the indicated proteins in the H2452 and H28 PM cell lines transfected with a scrambled control (sgCtrl) or NF2-targeting sgRNAs (sgNF2-1, sgNF2-2). The data are presented as the mean ± S.D.(n=3). One-way ANOVA with multiple comparisons was used for statistical analysis.

(D, E) Quantification data from immunoblots of the indicated proteins in normal mesothelial cells (Met-5A) and PM cell lines (NF2 wild-type: H28 and H2452; NF2 mutant: H2052). The data are presented as the mean ± S.D.(n=3). One-way ANOVA with multiple comparison was used for statistical analysis.

(F) Quantification data from immunoblots of the indicated proteins from H2452 and H28 sgCtrl and NF2-KO (sgNF2-1) PM cell lines treated with the indicated doses of the DHODH inhibitor Brequinar for 48h. The data are presented as the mean ± S.D.(n=3). Two-way ANOVA with multiple comparisons was used for statistical analysis.

(G) Quantification data from immunoblots of the indicated proteins in H2452 NF2-KO (sgNF2-1) cells transfected with siRNA targeting YAP (left) or treated with the specific YAP-TEAD inhibitor 1 (Peptide 17; right) for 48h. The data are presented as the mean ± S.D.(n=3). A two-tailed unpaired t test was used for statistical analysis.

(H, I) Quantification data from immunoblots of NF2 in the indicated populations after transfection with siRNA targeting CAD or DHODH. The data are presented as the mean ± S.D.(n=3). Two-way ANOVA with multiple comparisons was used for statistical analysis.

(J, K) Quantification data from immunoblots of the indicated proteins in H2452 sgNF2-1 (KO1) and sgNF2-2 (KO2) PM cells transfected with a negative control (NC) or sgRNA-resistant NF2 cDNA (OE). The data are presented as the mean ± S.D.(n=3). One-way ANOVA with multiple comparisons was used for statistical analysis.

(L) Quantification data from immunoblots of the indicated proteins in PDCLs. The data are presented as the mean ± S.D.(n=3).

Appendix Figure S6

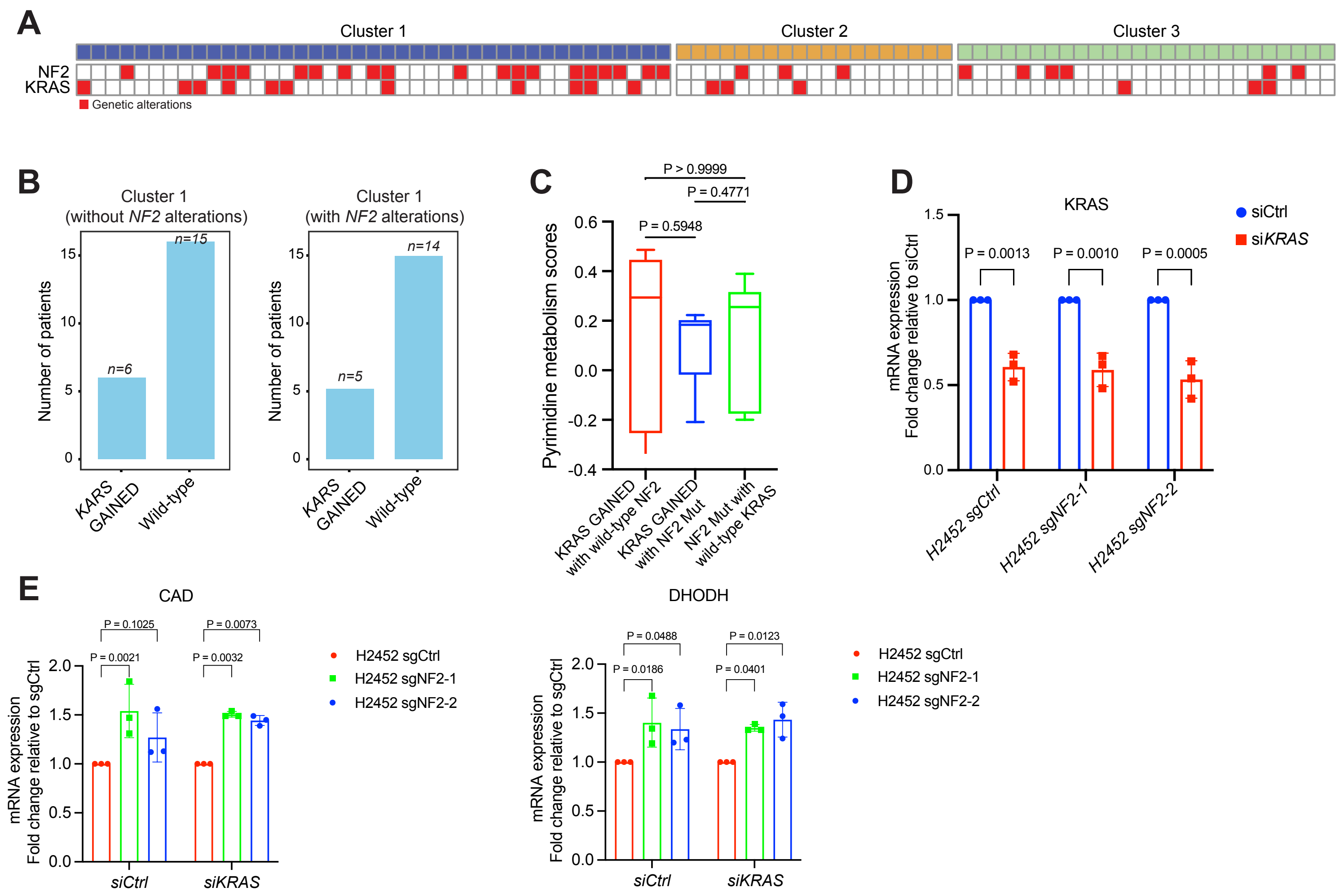

Appendix Figure S6: *NF2* loss-driven activated pyrimidine metabolism is independently of *KRAS* signaling.

(A) The genetic alterations of *NF2* and *KRAS* among metabolic subgroups in TCGA-MESO cohort (n=87).Genetic status of *KRAS* is defined based on the study (Marazioti et al., 2022).

(B) The number of patients exhibiting *KRAS* mutations or wild type within Cluster 1, categorized by the absence (left) or presence (right) of *NF2* alterations.

(C) The difference of py rimidine metabolism GSVA scores in the indicated groups. One-way ANOVA with multiple comparisions.

(D, E) mRNA of *KRAS*, *CAD*, and *DHODH* in the indicated groups transfected with nontargeting control or siRNA targeting KRAS for 72h. The data are presented as the mean ± S.D. (n=3). Two-way ANOVA with multiple comparisions.

Appendix Figure S7

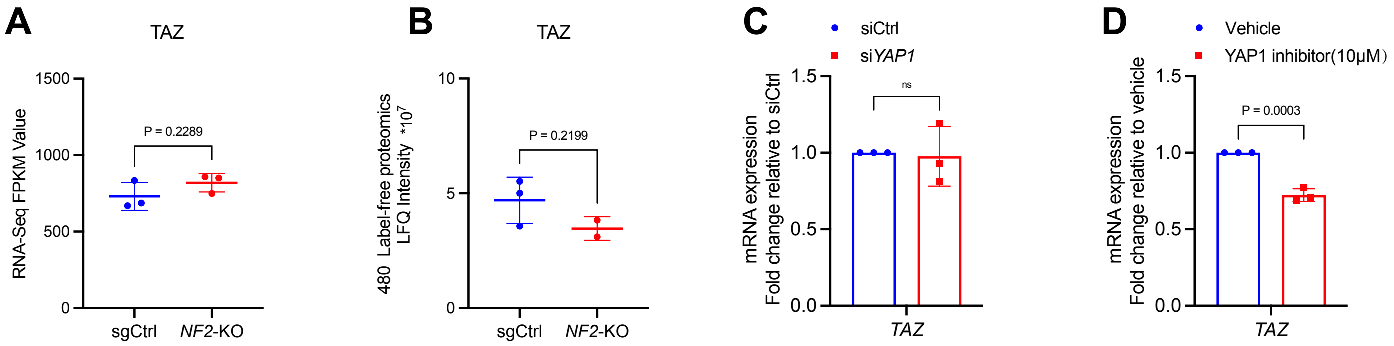

Appendix Figure S7: TAZ expression is not affected by NF2 loss or direct YAP manipulations.

(A, B) The difference in FPKM values from RNA-seq (A) and LFQ intensities from 480 label-free proteomics (B) of TAZ between H2452 sg*NF2* and sgCtrl groups.

(C, D) mRNA expression of TAZ in H2452 NF2-KO cells transfected with the indicated siRNAs (C) or treated with the YAP-TEAD inhibitor 1 (Peptide 17) for 48 h (D). The data are presented as the mean ± S.D. A two-tailed unpaired t test was used for comparison.
