# Supplementary figures and images for: De novo pyrimidine synthesis is a collateral metabolic vulnerability in NF2-deficient mesothelioma
Source: EMBO Mol Med. 2025 Jul 24;17(9):2258–98. doi: 10.1038/s44321-025-00278-4 (PMC12423300; doi:10.1038/s44321-025-00278-4)

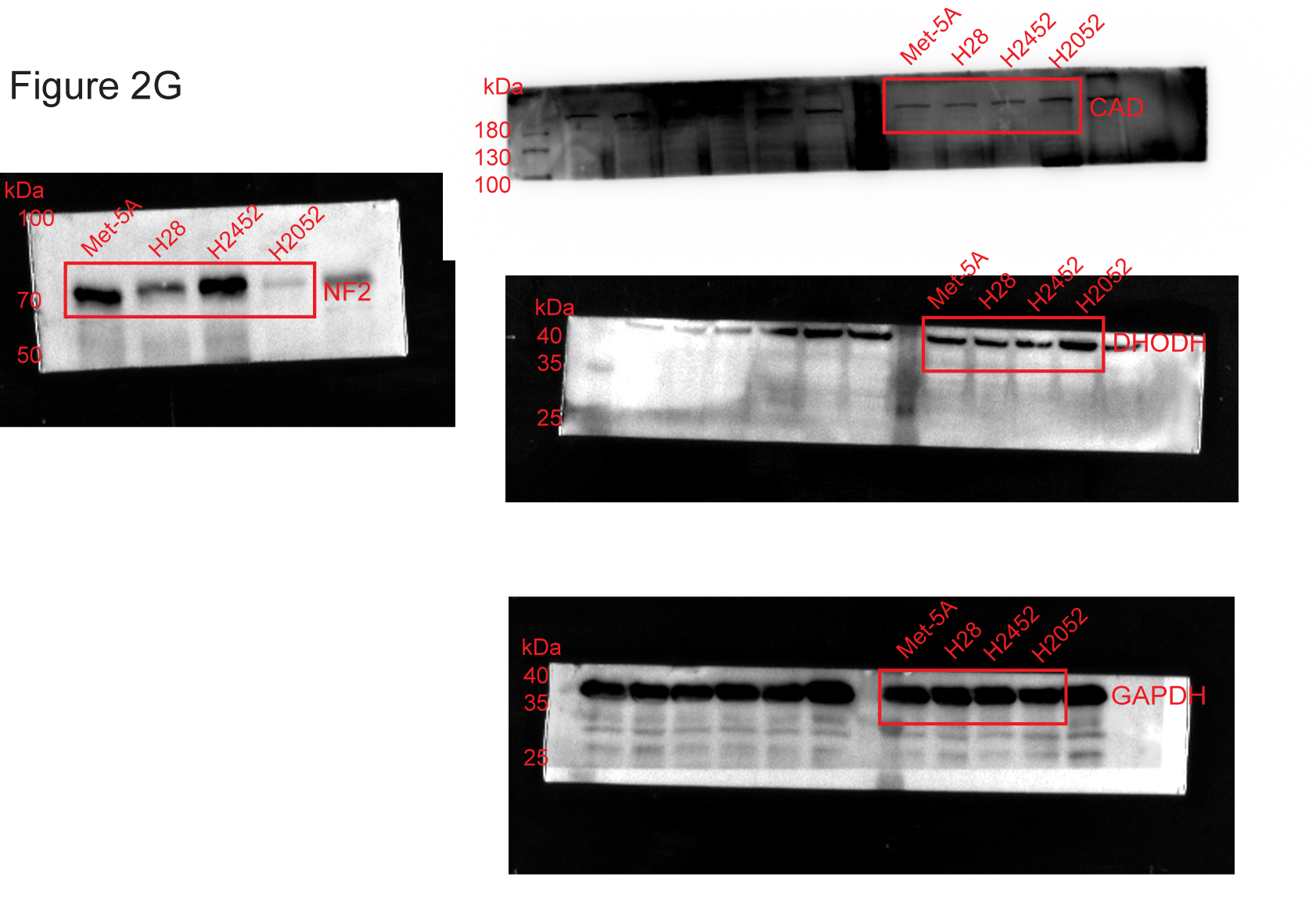

Supplement: Supplementary file 26 — Source data Fig. 2 [file 44321_2025_278_MOESM26_ESM.zip › Figure 2/2G/2G.tif]

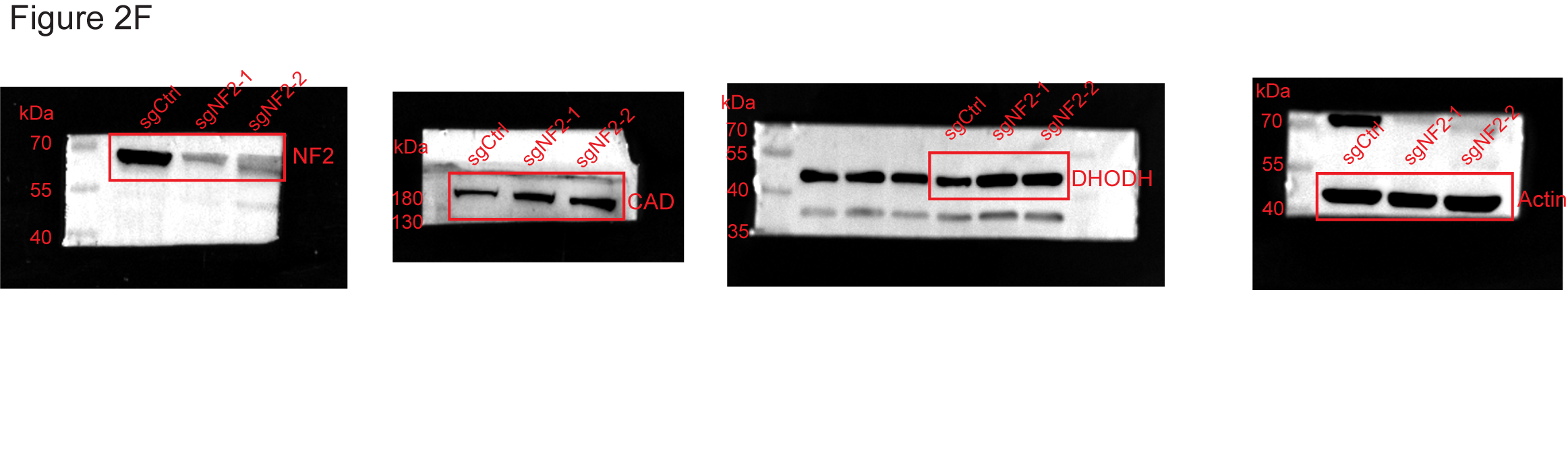

Supplement: Supplementary file 26 — Source data Fig. 2 [file 44321_2025_278_MOESM26_ESM.zip › Figure 2/2F/Figure 2F.tif]

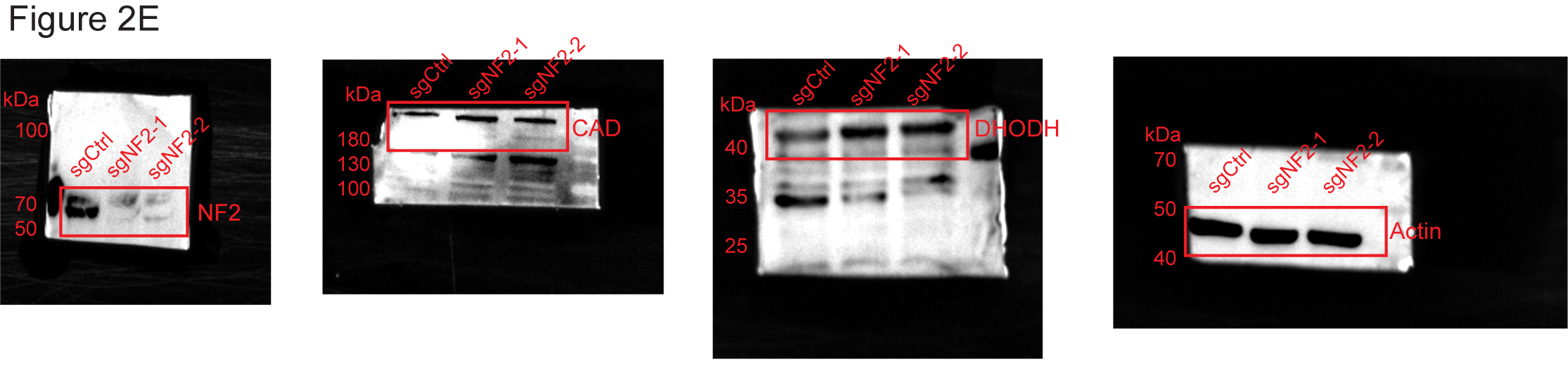

Supplement: Supplementary file 26 — Source data Fig. 2 [file 44321_2025_278_MOESM26_ESM.zip › Figure 2/2E/Figure 2E.tif]

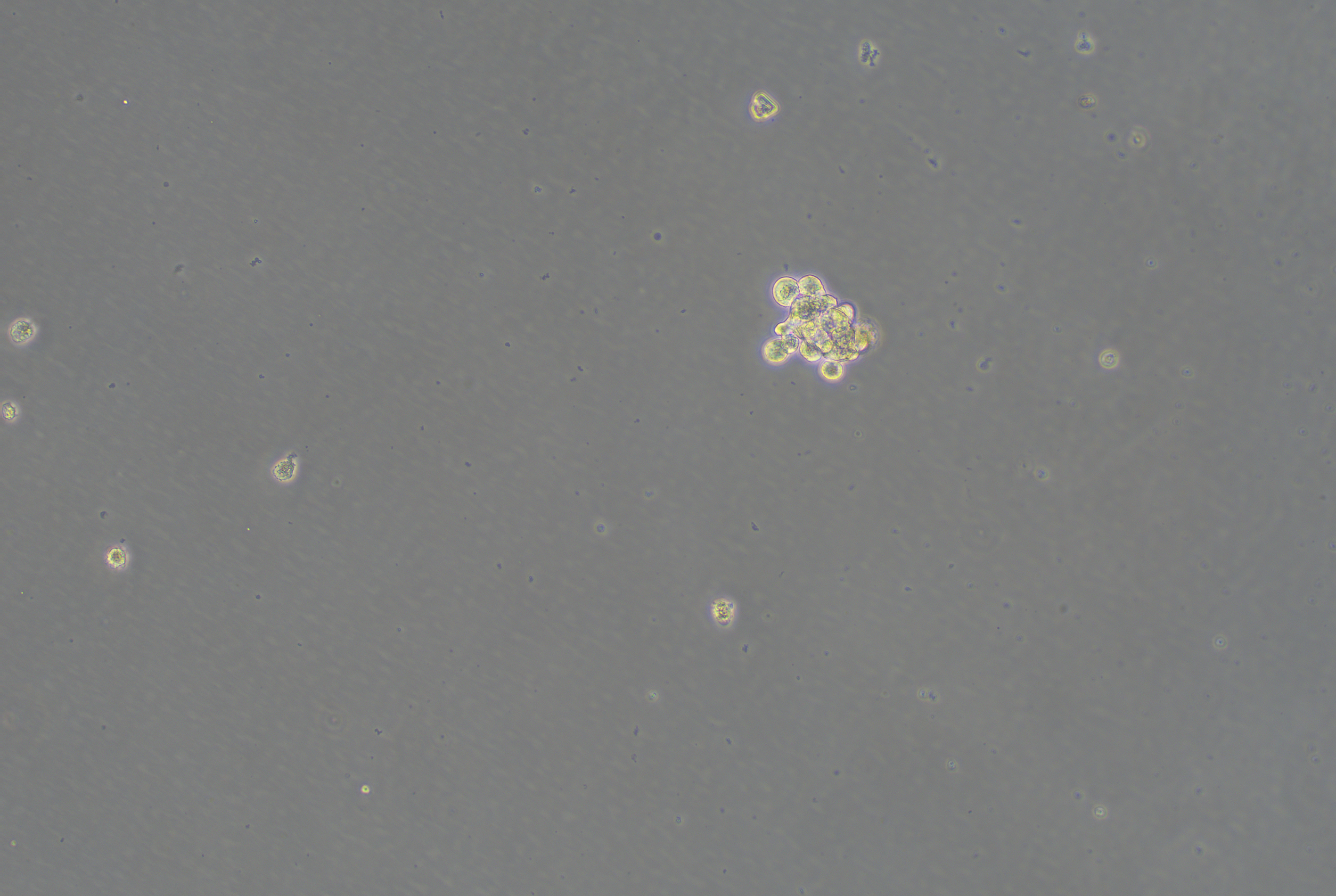

Supplement: Supplementary file 27 — Source data Fig. 3 [file 44321_2025_278_MOESM27_ESM.zip › Figure 3/3F/H2452 sgNF2-1_DHODHi 1uM.tif]

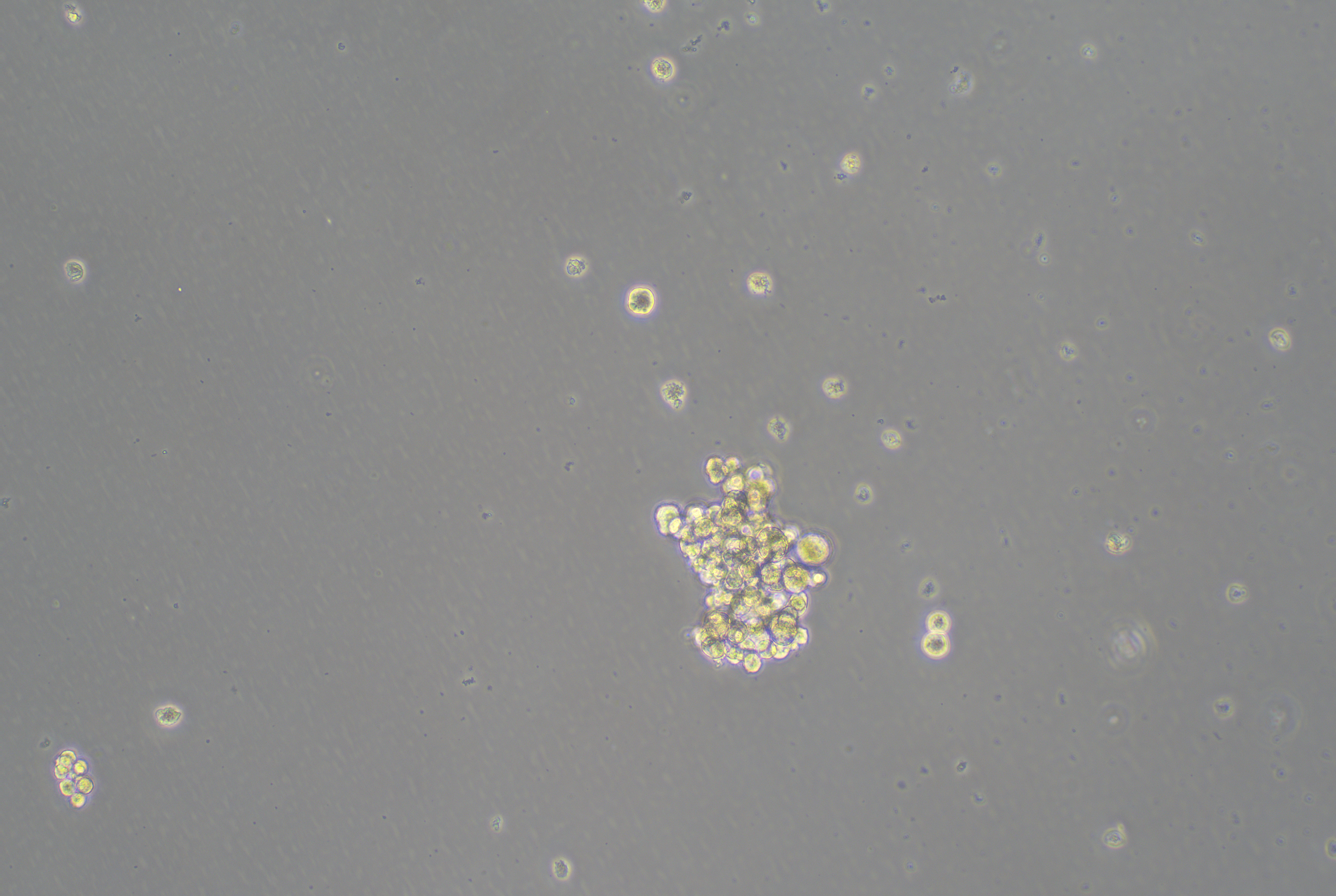

Supplement: Supplementary file 27 — Source data Fig. 3 [file 44321_2025_278_MOESM27_ESM.zip › Figure 3/3F/H2452 sgCtrl_DHODHi 1uM.tif]

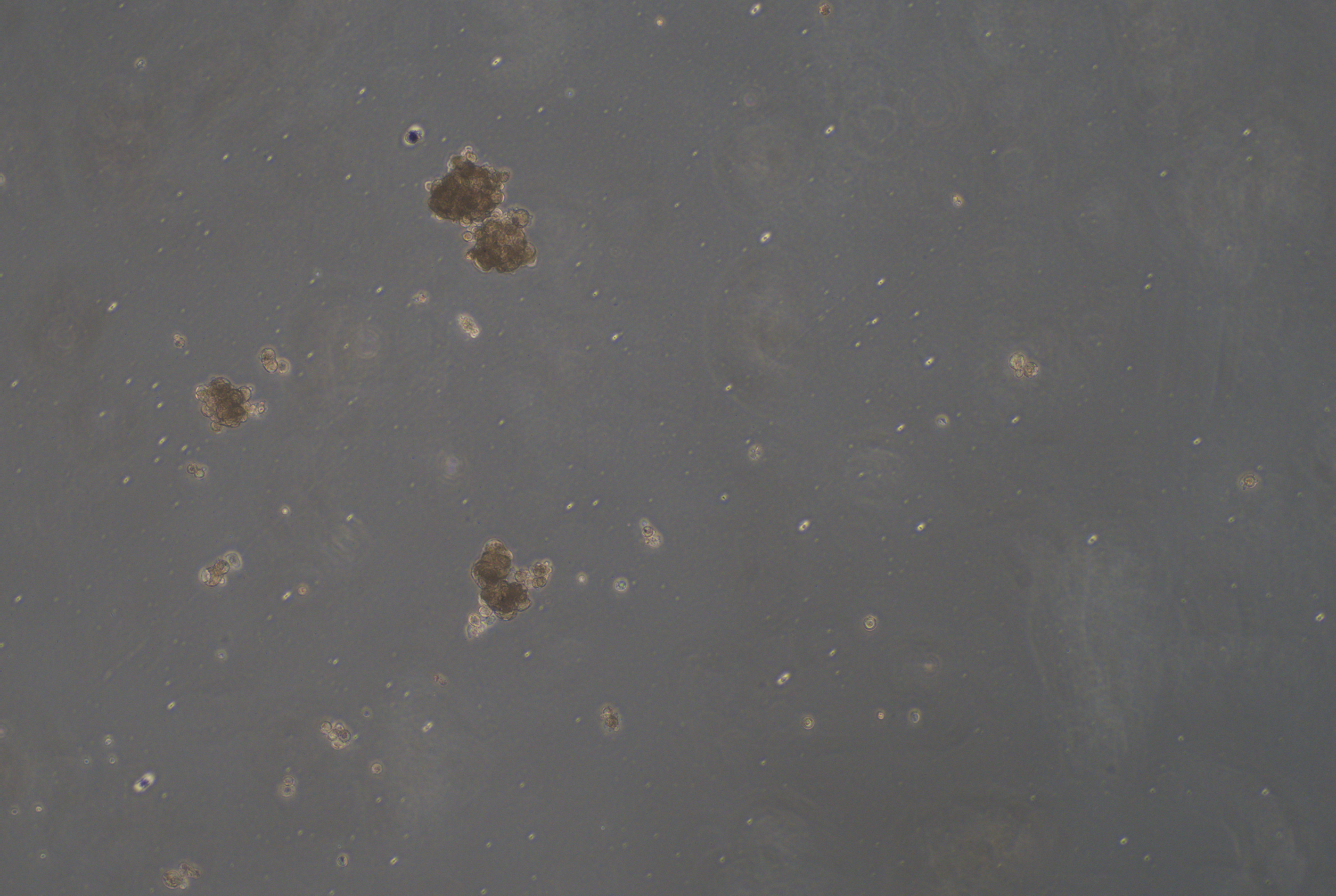

Supplement: Supplementary file 27 — Source data Fig. 3 [file 44321_2025_278_MOESM27_ESM.zip › Figure 3/3F/H28 sgCtrl DHODHi 1uM.tif]

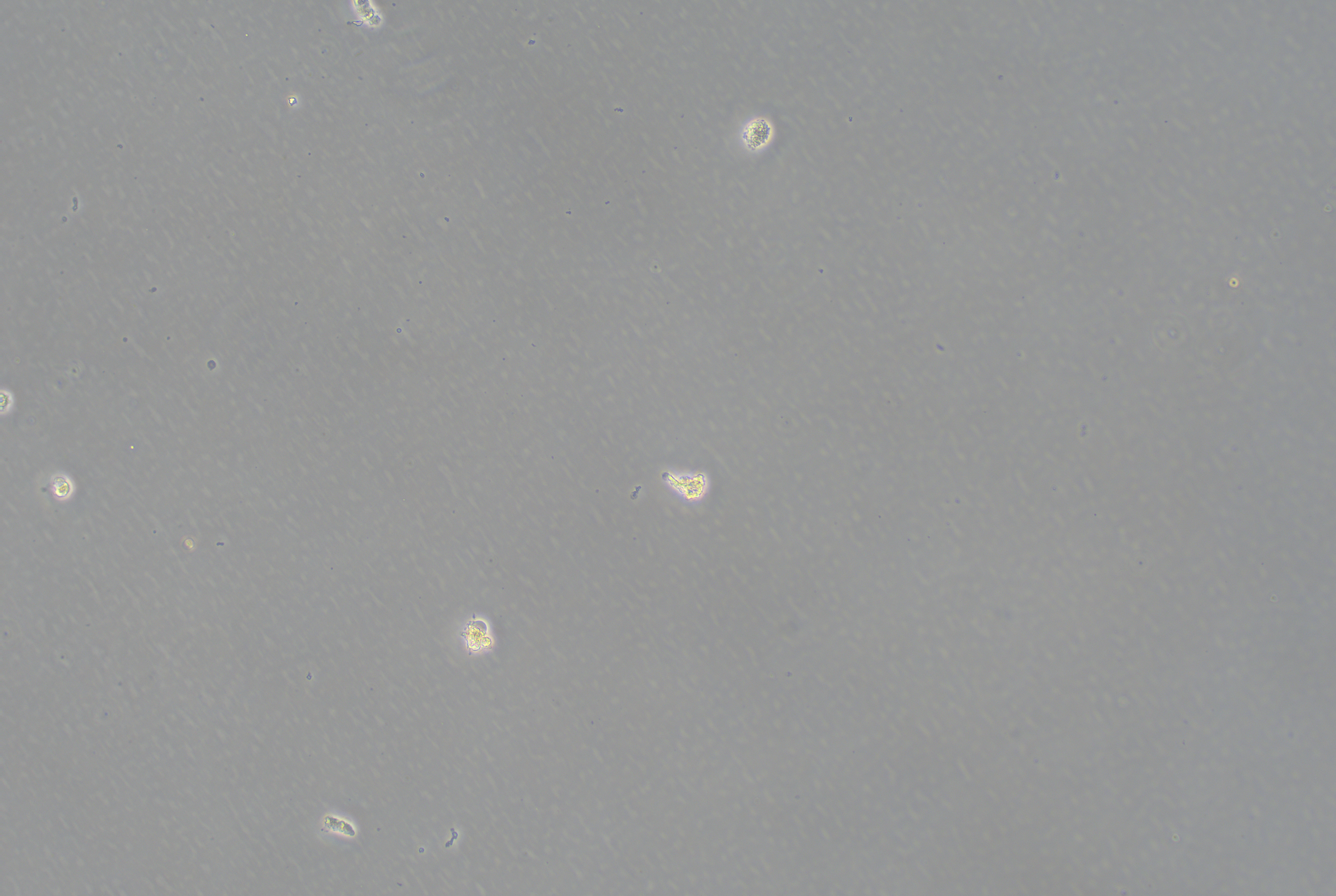

Supplement: Supplementary file 27 — Source data Fig. 3 [file 44321_2025_278_MOESM27_ESM.zip › Figure 3/3F/H2452 sgNF2-2_DHODHi 1uM.tif]

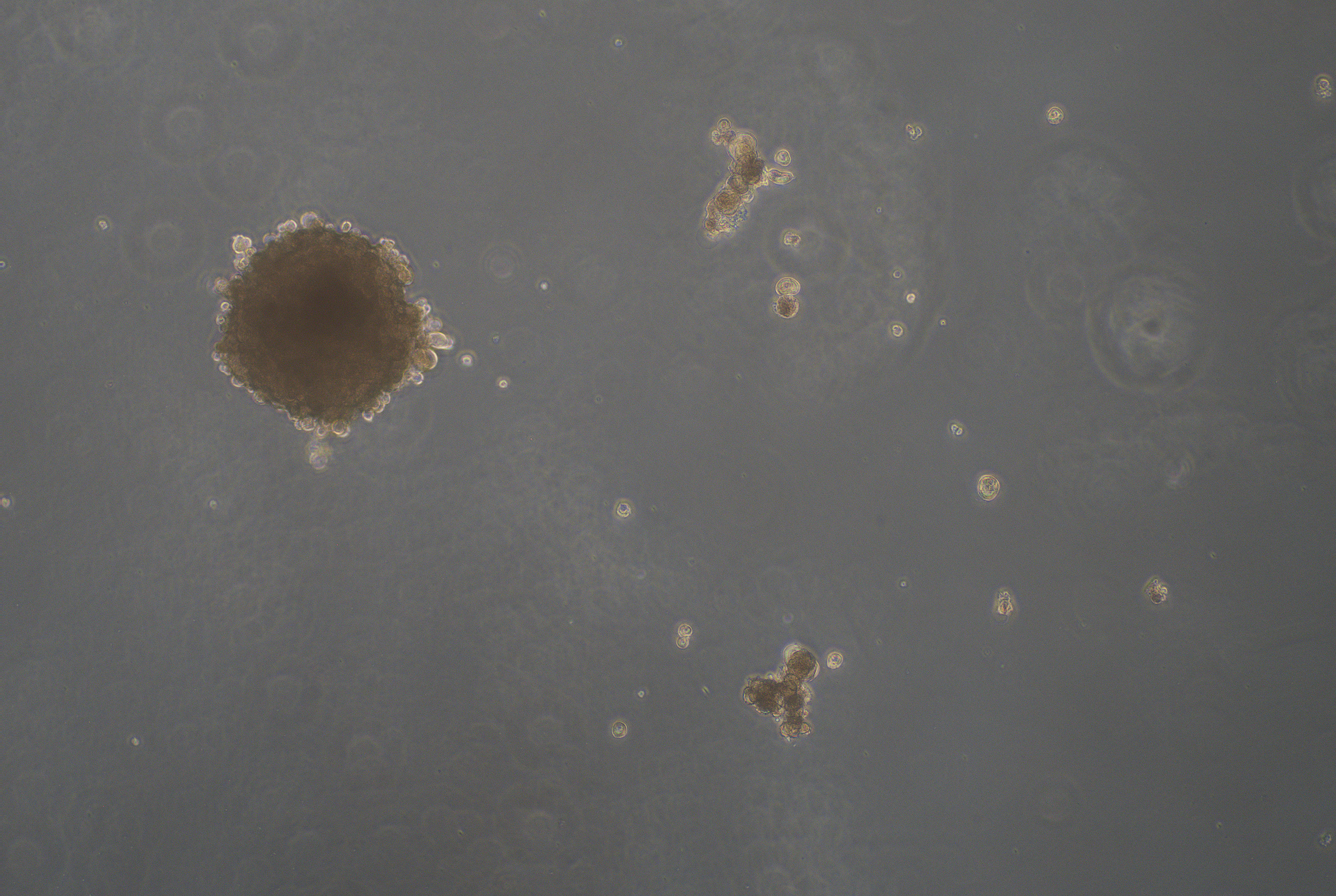

Supplement: Supplementary file 27 — Source data Fig. 3 [file 44321_2025_278_MOESM27_ESM.zip › Figure 3/3F/H28 sgNF2-2_Vehicle.tif]

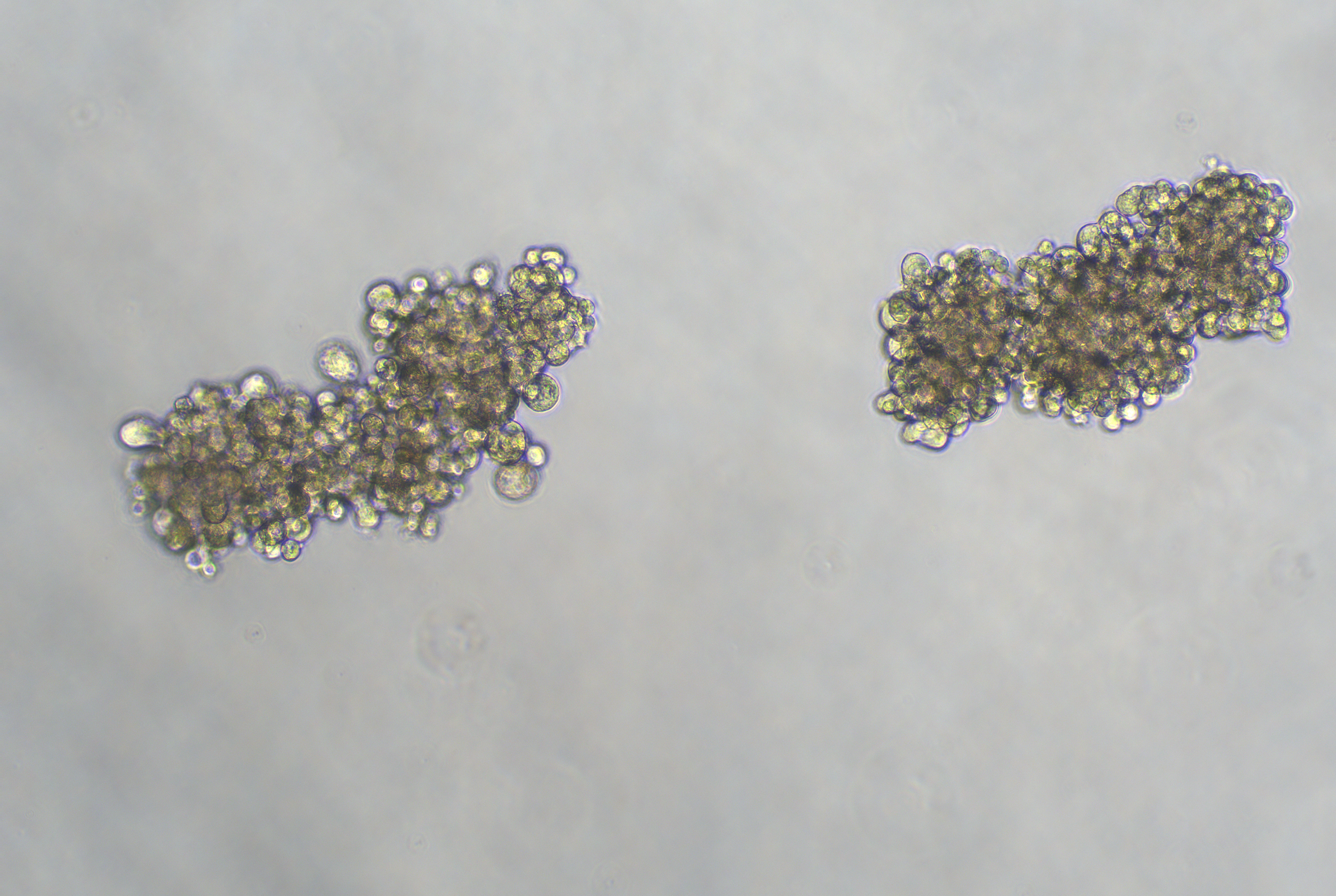

Supplement: Supplementary file 27 — Source data Fig. 3 [file 44321_2025_278_MOESM27_ESM.zip › Figure 3/3F/H2452 sgNF2-1_Vehicle.tif]

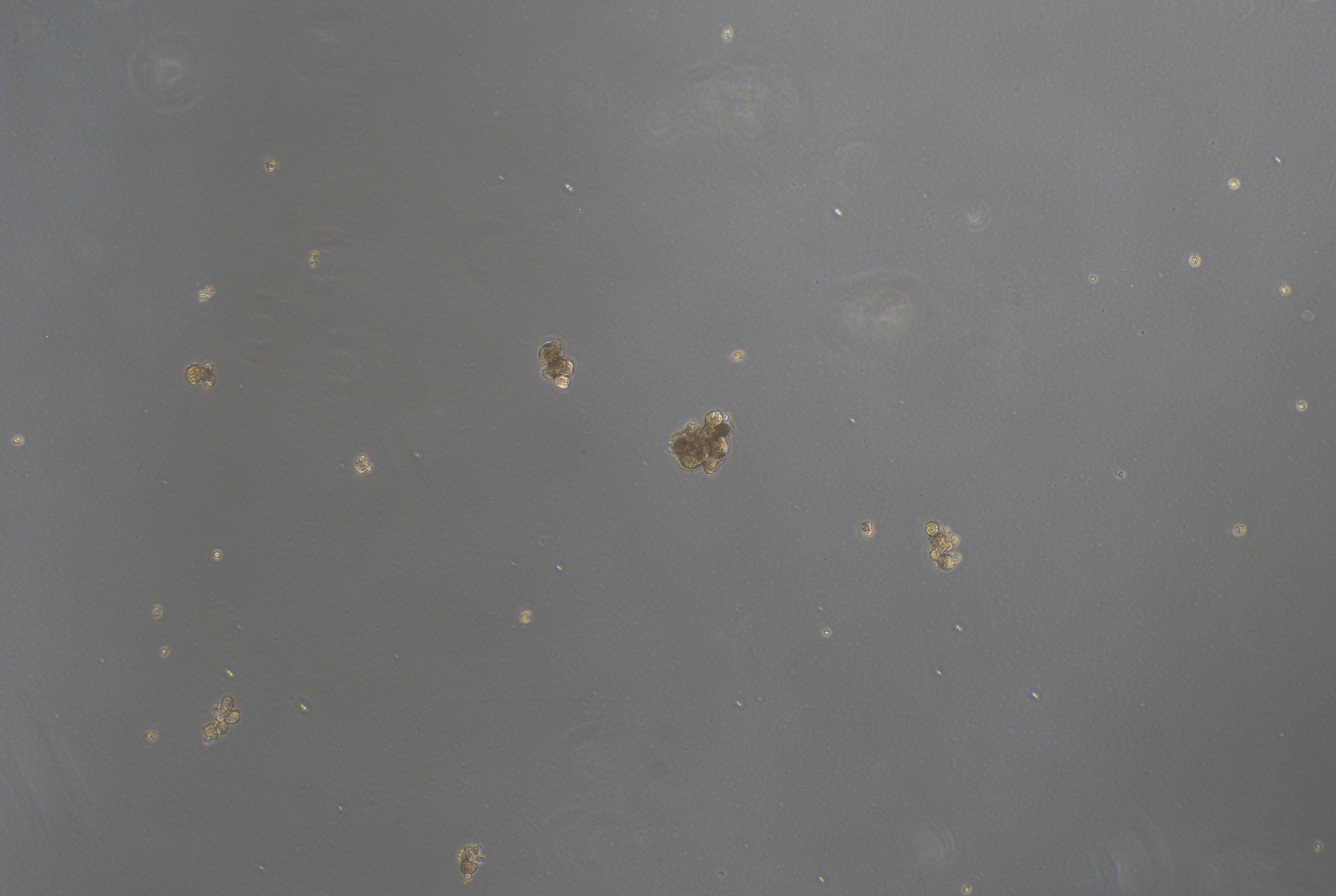

Supplement: Supplementary file 27 — Source data Fig. 3 [file 44321_2025_278_MOESM27_ESM.zip › Figure 3/3F/H28 sgNF2-2 DHODHi 1uM.tif]

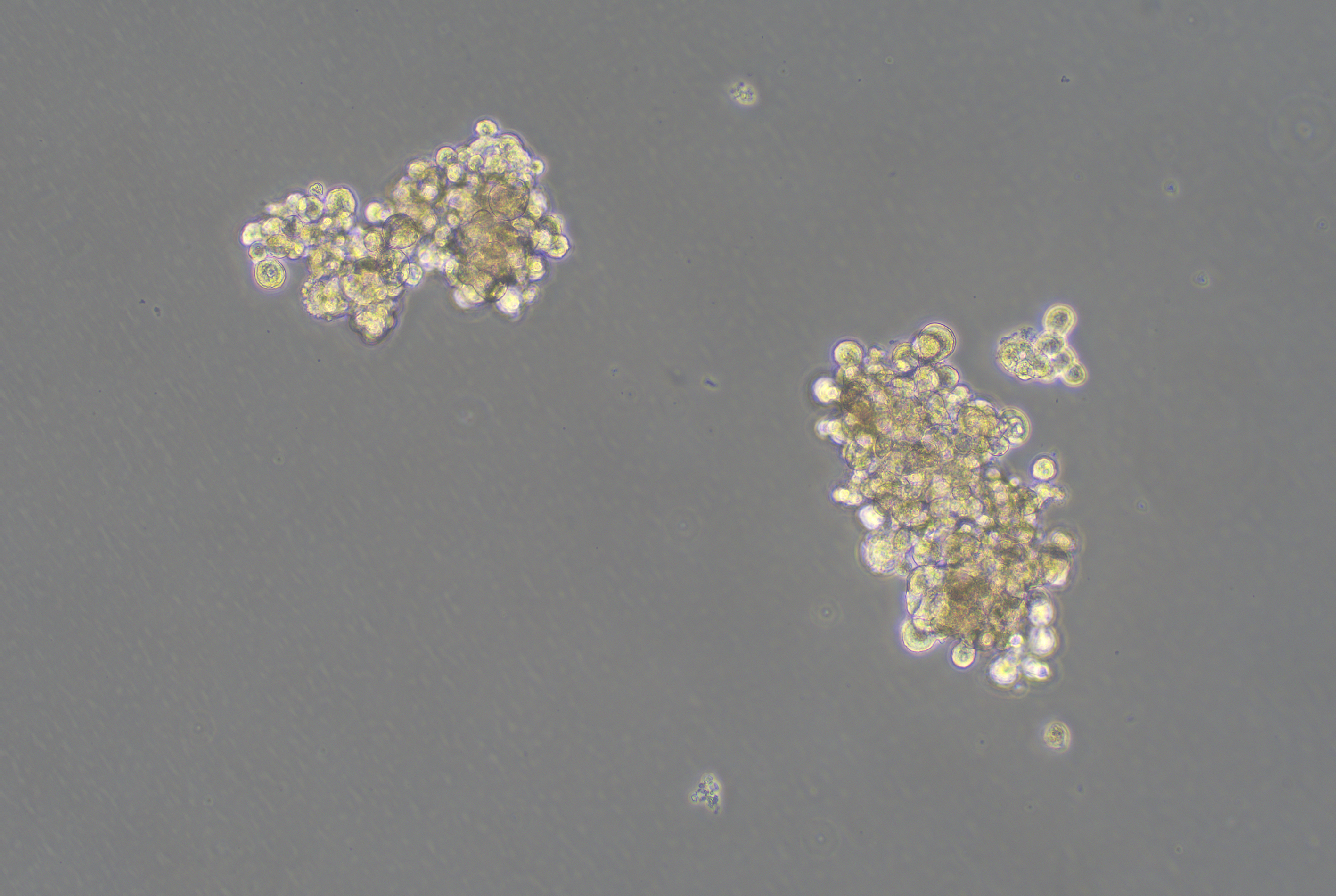

Supplement: Supplementary file 27 — Source data Fig. 3 [file 44321_2025_278_MOESM27_ESM.zip › Figure 3/3F/H2452 sgNF2-2_Vehicle.tif]

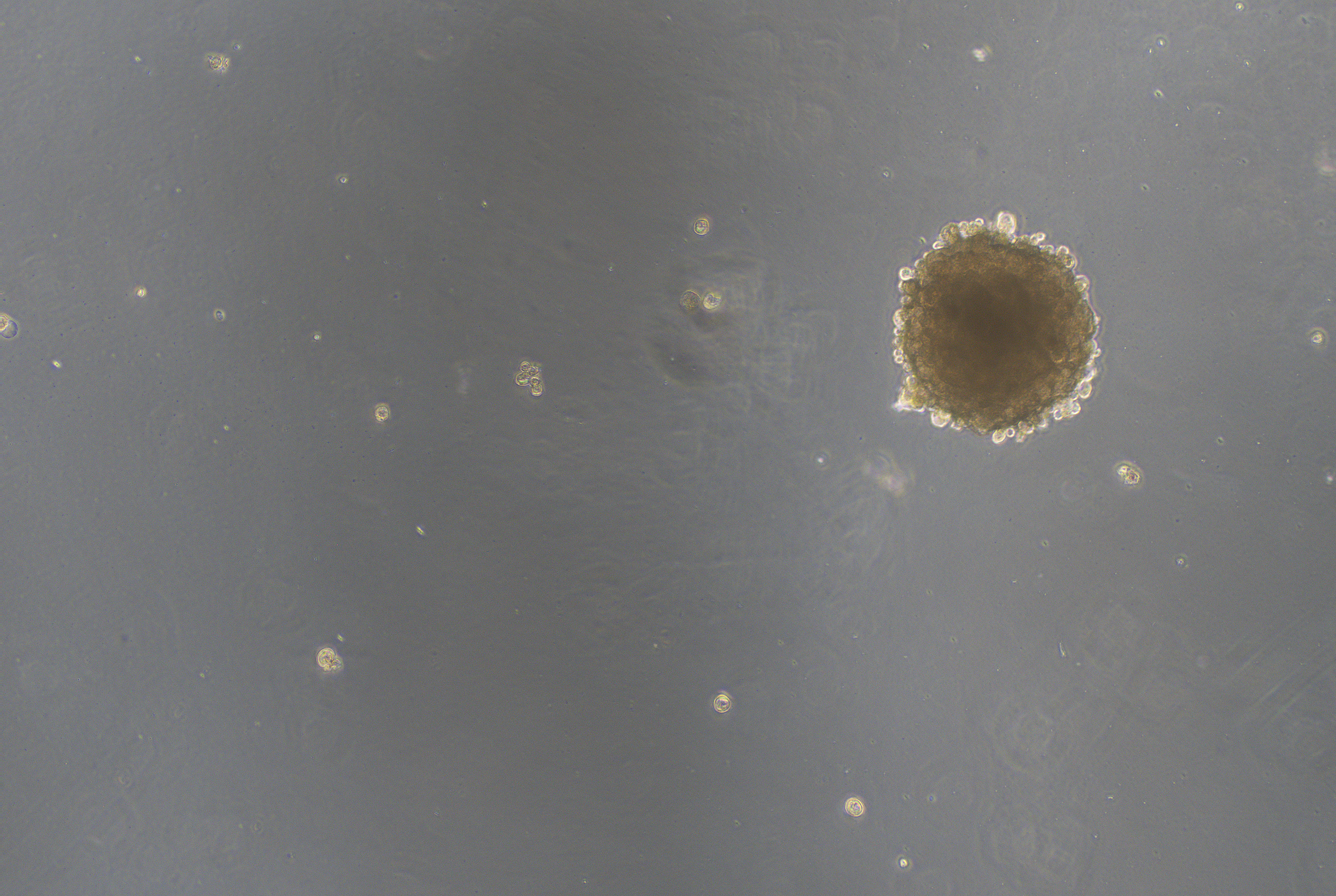

Supplement: Supplementary file 27 — Source data Fig. 3 [file 44321_2025_278_MOESM27_ESM.zip › Figure 3/3F/H28 sgNF2-1_Vehicle.tif]

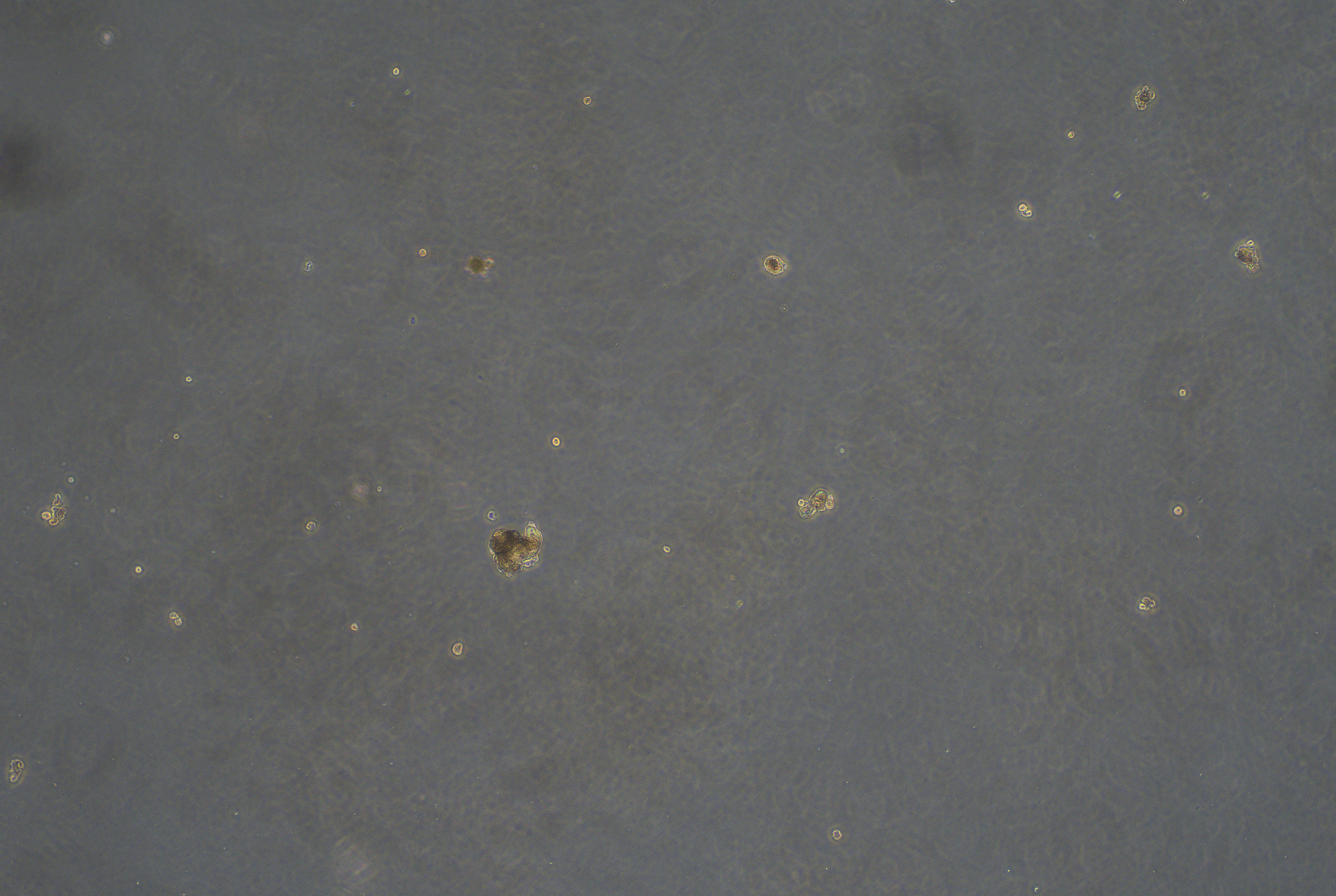

Supplement: Supplementary file 27 — Source data Fig. 3 [file 44321_2025_278_MOESM27_ESM.zip › Figure 3/3F/H28 sgNF2-1 DHODHi 1uM.tif]

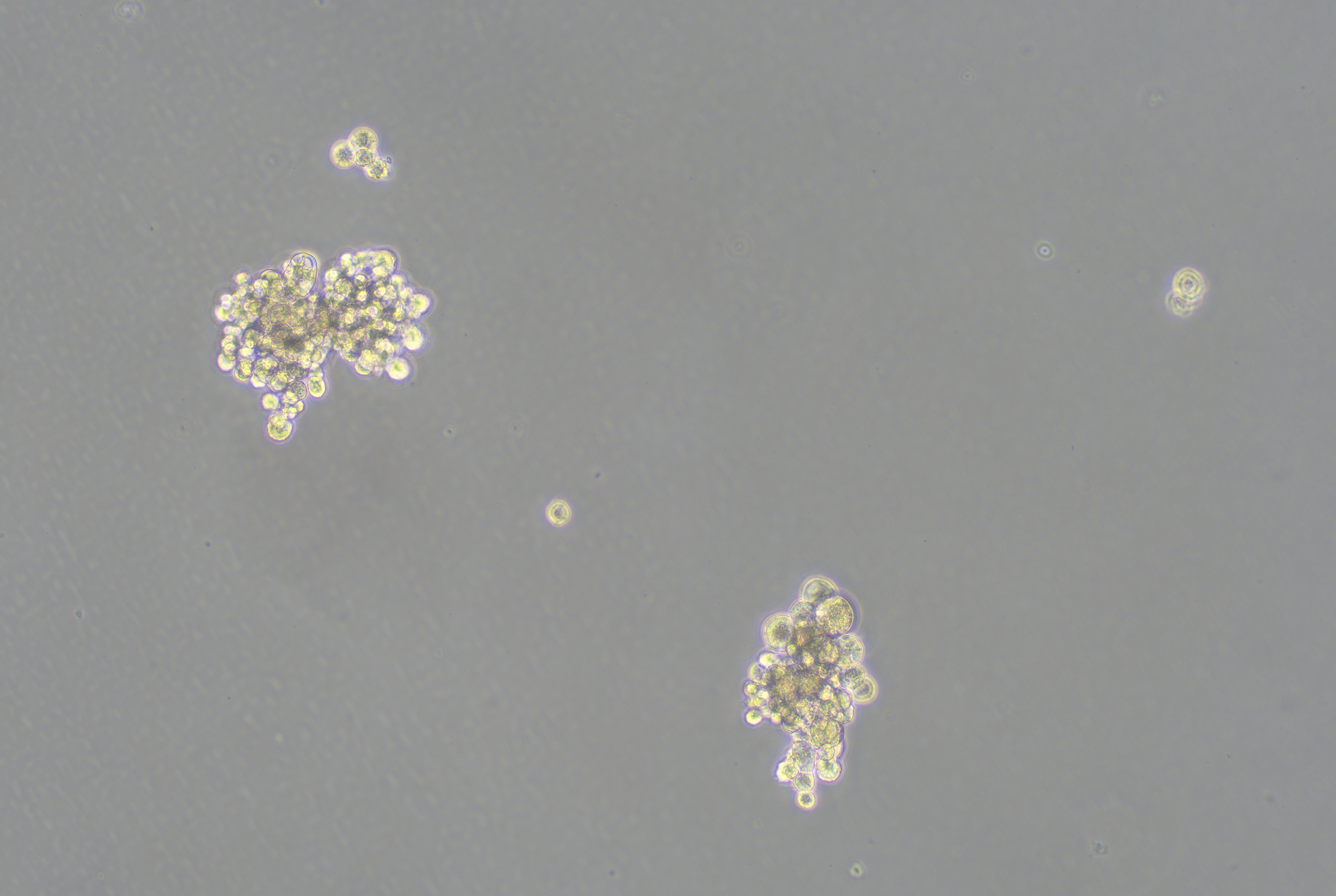

Supplement: Supplementary file 27 — Source data Fig. 3 [file 44321_2025_278_MOESM27_ESM.zip › Figure 3/3F/H2452 sgCtrl_Vehicle.tif]

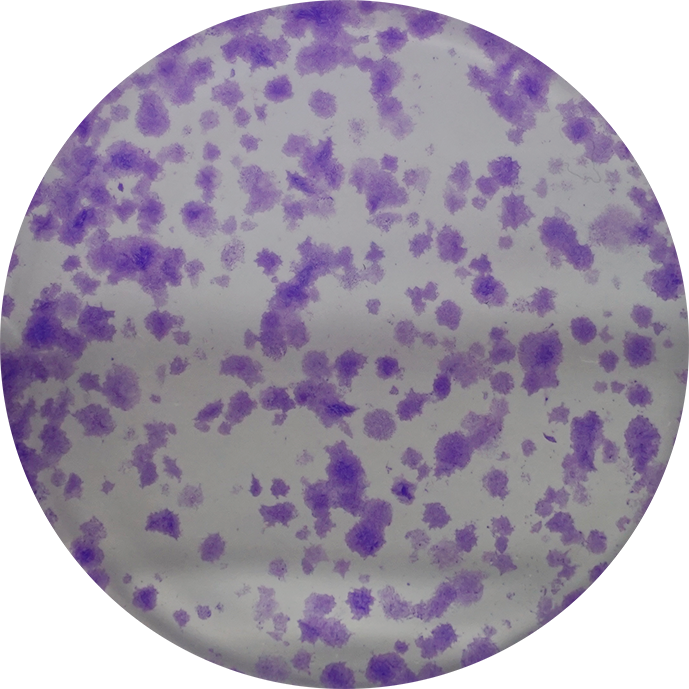

Supplement: Supplementary file 27 — Source data Fig. 3 [file 44321_2025_278_MOESM27_ESM.zip › Figure 3/3D/3D_top/H2452 NF2 KO2.tif]

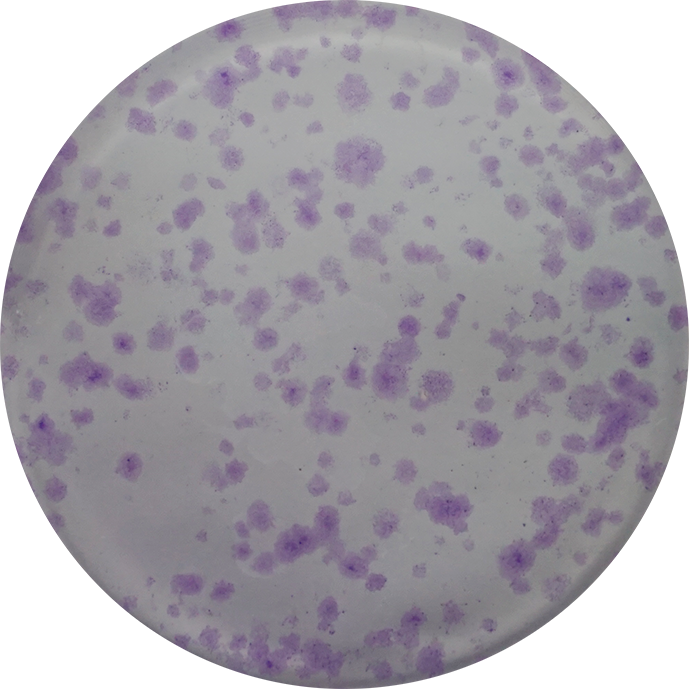

Supplement: Supplementary file 27 — Source data Fig. 3 [file 44321_2025_278_MOESM27_ESM.zip › Figure 3/3D/3D_top/H2452 NF2 KO2 DHODHi.tif]

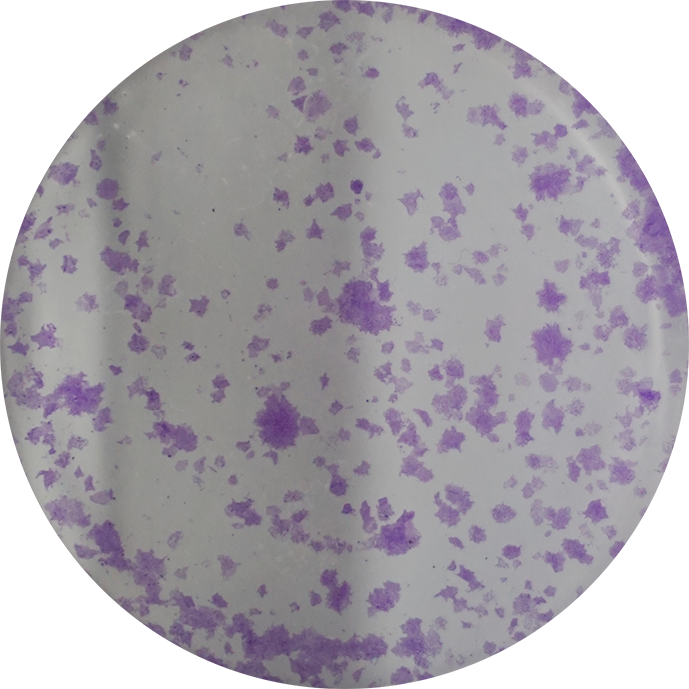

Supplement: Supplementary file 27 — Source data Fig. 3 [file 44321_2025_278_MOESM27_ESM.zip › Figure 3/3D/3D_top/H2452 sgCtrl.tif]

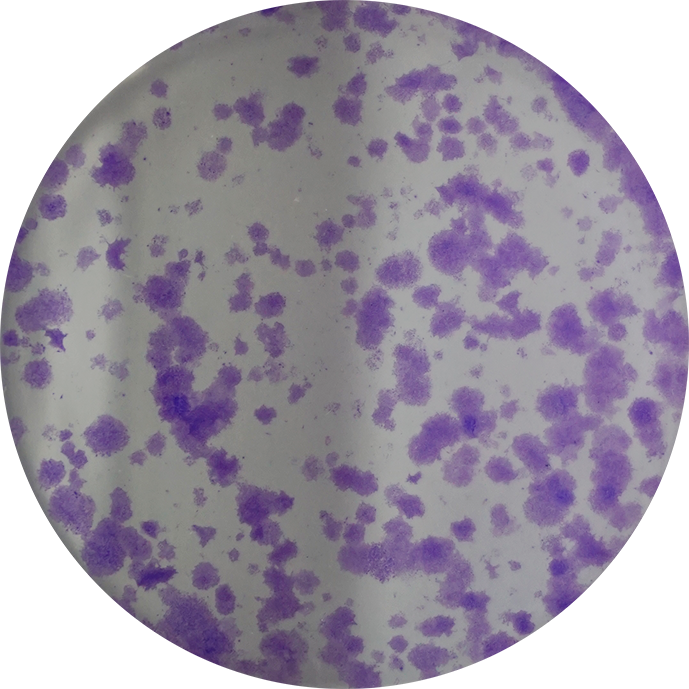

Supplement: Supplementary file 27 — Source data Fig. 3 [file 44321_2025_278_MOESM27_ESM.zip › Figure 3/3D/3D_top/H2452 NF2 KO1.tif]

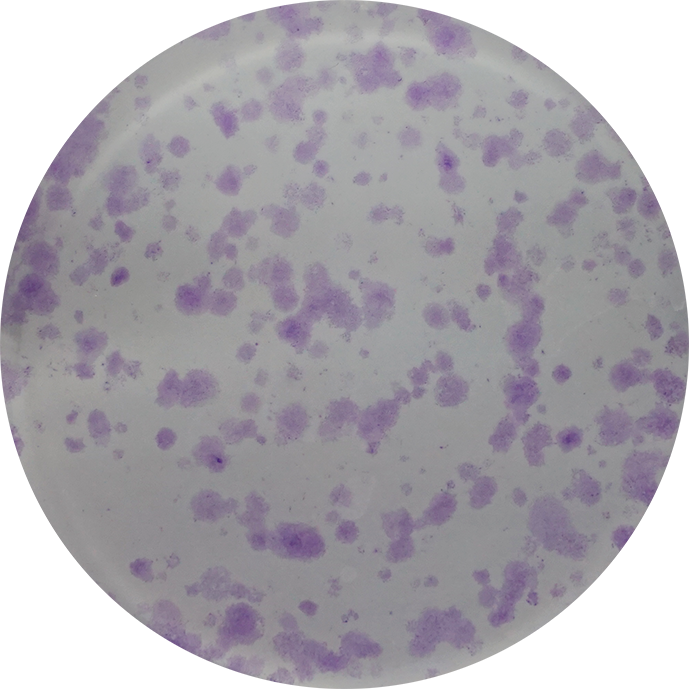

Supplement: Supplementary file 27 — Source data Fig. 3 [file 44321_2025_278_MOESM27_ESM.zip › Figure 3/3D/3D_top/H2452 NF2 KO1 DHODHi.tif]

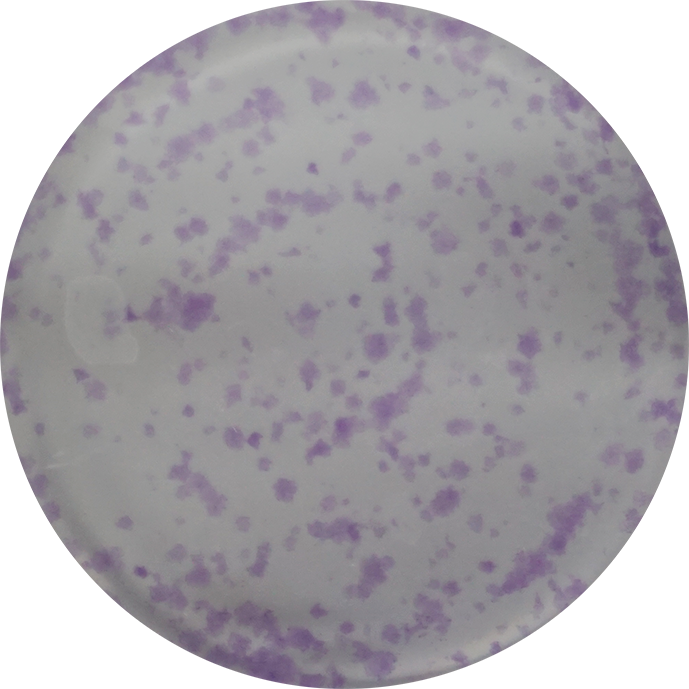

Supplement: Supplementary file 27 — Source data Fig. 3 [file 44321_2025_278_MOESM27_ESM.zip › Figure 3/3D/3D_top/H2452 sgCtrl DHODHi 1uM.tif]

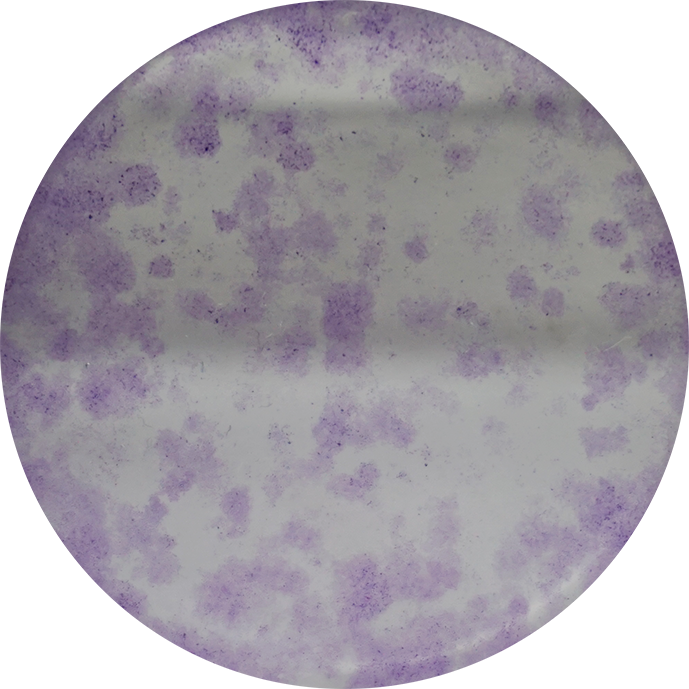

Supplement: Supplementary file 27 — Source data Fig. 3 [file 44321_2025_278_MOESM27_ESM.zip › Figure 3/3D/3D_down/H28 sgCtrl.tif]

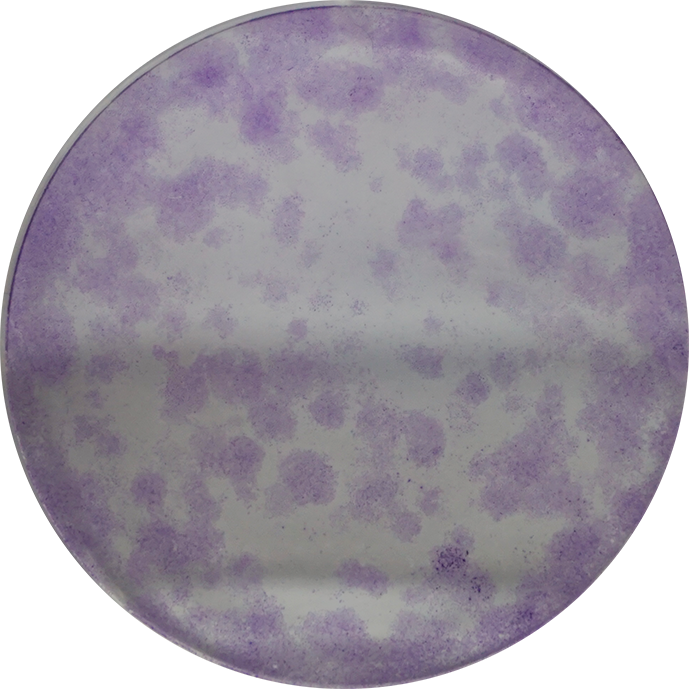

Supplement: Supplementary file 27 — Source data Fig. 3 [file 44321_2025_278_MOESM27_ESM.zip › Figure 3/3D/3D_down/H28 NF2-KO2.tif]

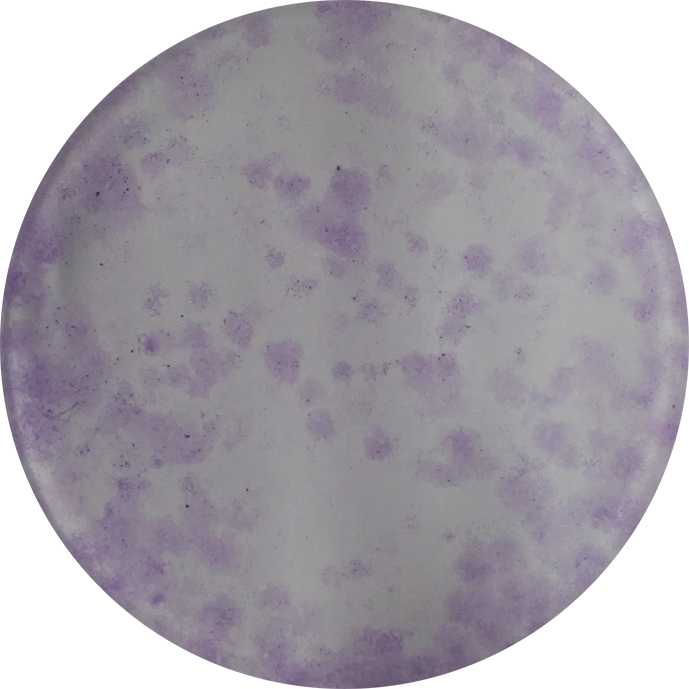

Supplement: Supplementary file 27 — Source data Fig. 3 [file 44321_2025_278_MOESM27_ESM.zip › Figure 3/3D/3D_down/H28 sgCtrl DHODHi 1uM.tif]

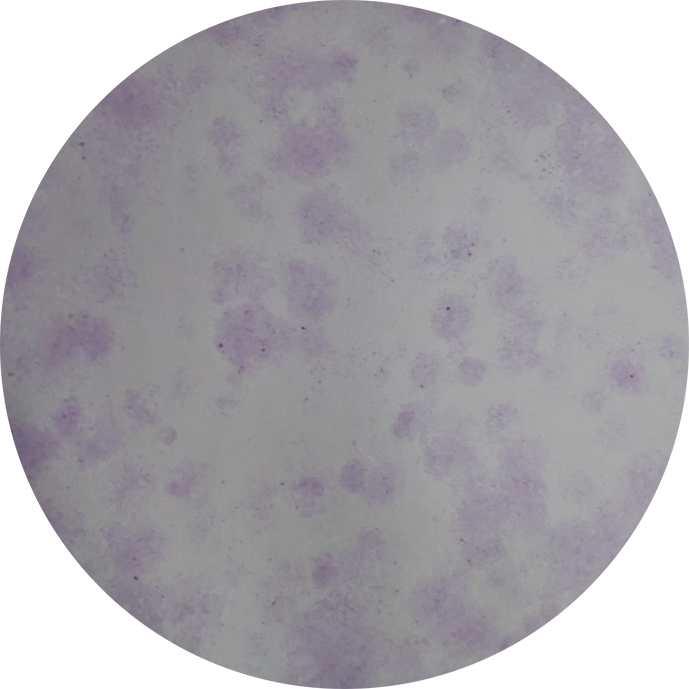

Supplement: Supplementary file 27 — Source data Fig. 3 [file 44321_2025_278_MOESM27_ESM.zip › Figure 3/3D/3D_down/H28 NF2-KO2 DHODHi.tif]

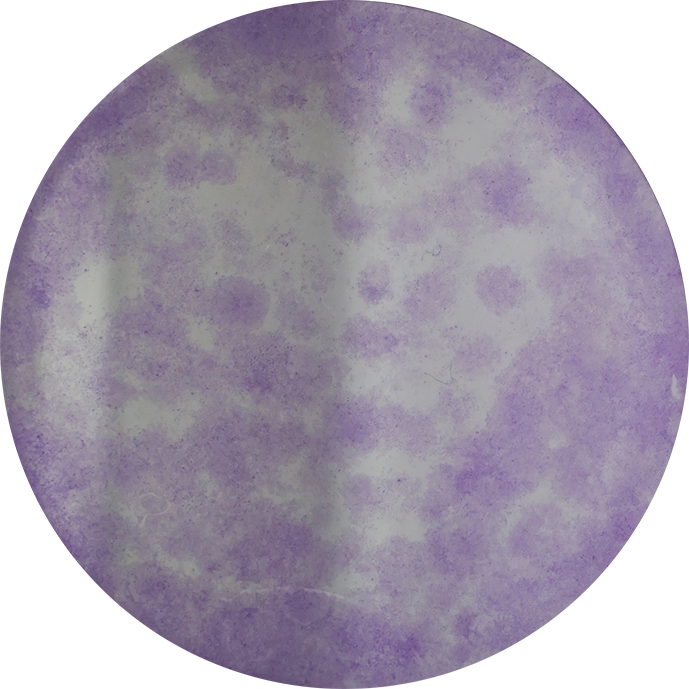

Supplement: Supplementary file 27 — Source data Fig. 3 [file 44321_2025_278_MOESM27_ESM.zip › Figure 3/3D/3D_down/H28 NF2 KO1.tif]

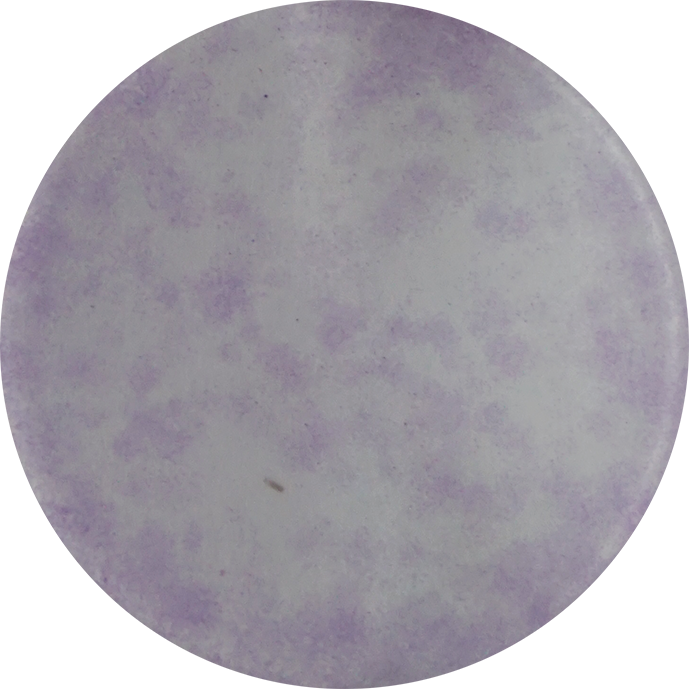

Supplement: Supplementary file 27 — Source data Fig. 3 [file 44321_2025_278_MOESM27_ESM.zip › Figure 3/3D/3D_down/H28 NF2-KO1 DHODHi.tif]

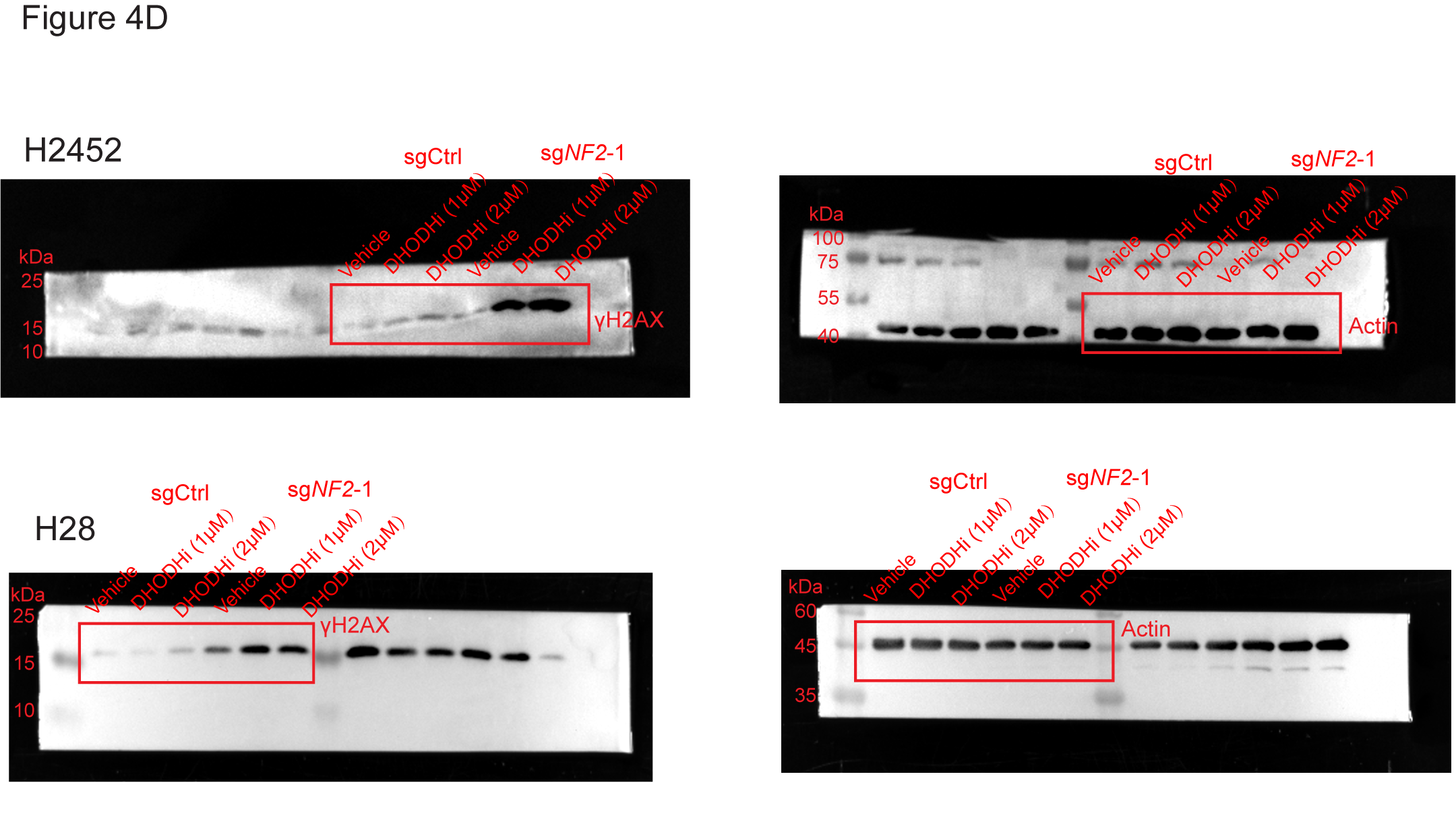

Supplement: Supplementary file 28 — Source data Fig. 4 [file 44321_2025_278_MOESM28_ESM.zip › Figure 4/4D/4D.tif]

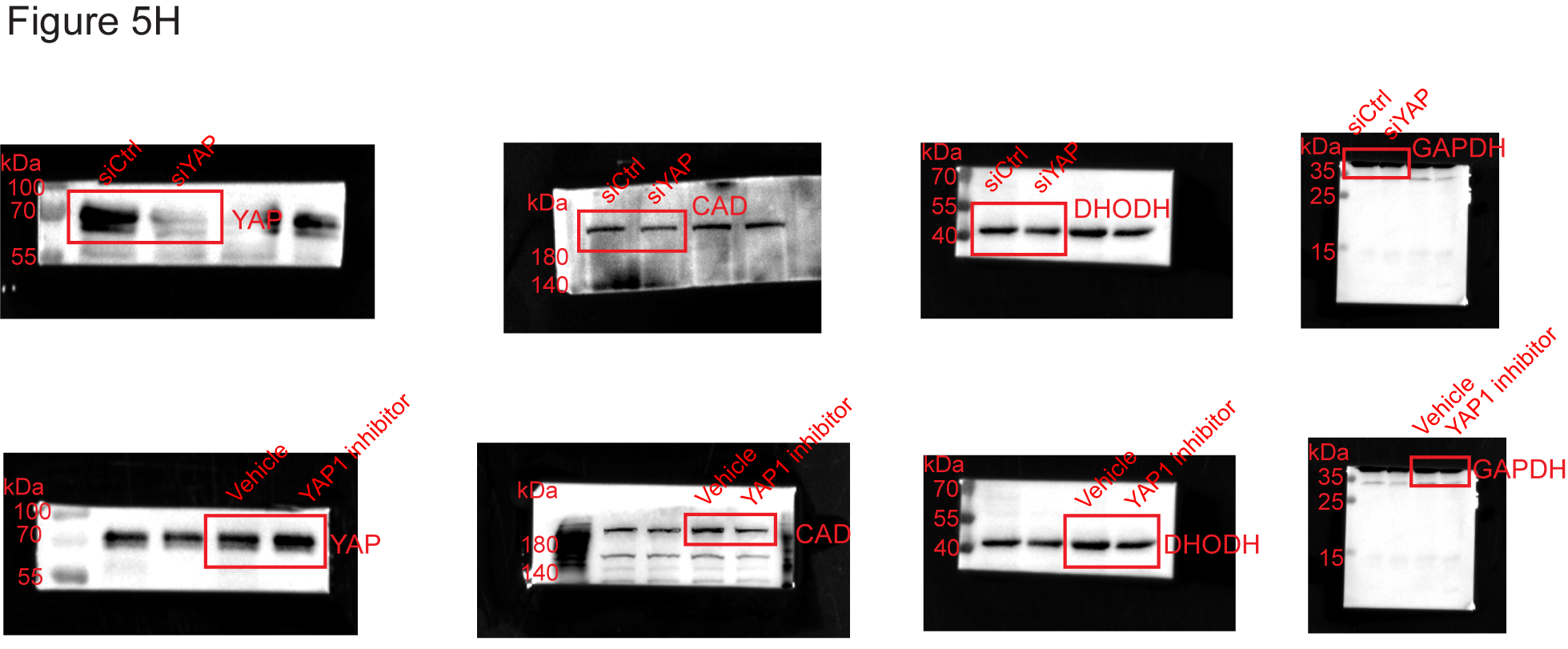

Supplement: Supplementary file 29 — Source data Fig. 5 [file 44321_2025_278_MOESM29_ESM.zip › Figure 5/5H/Figure 5H.tif]

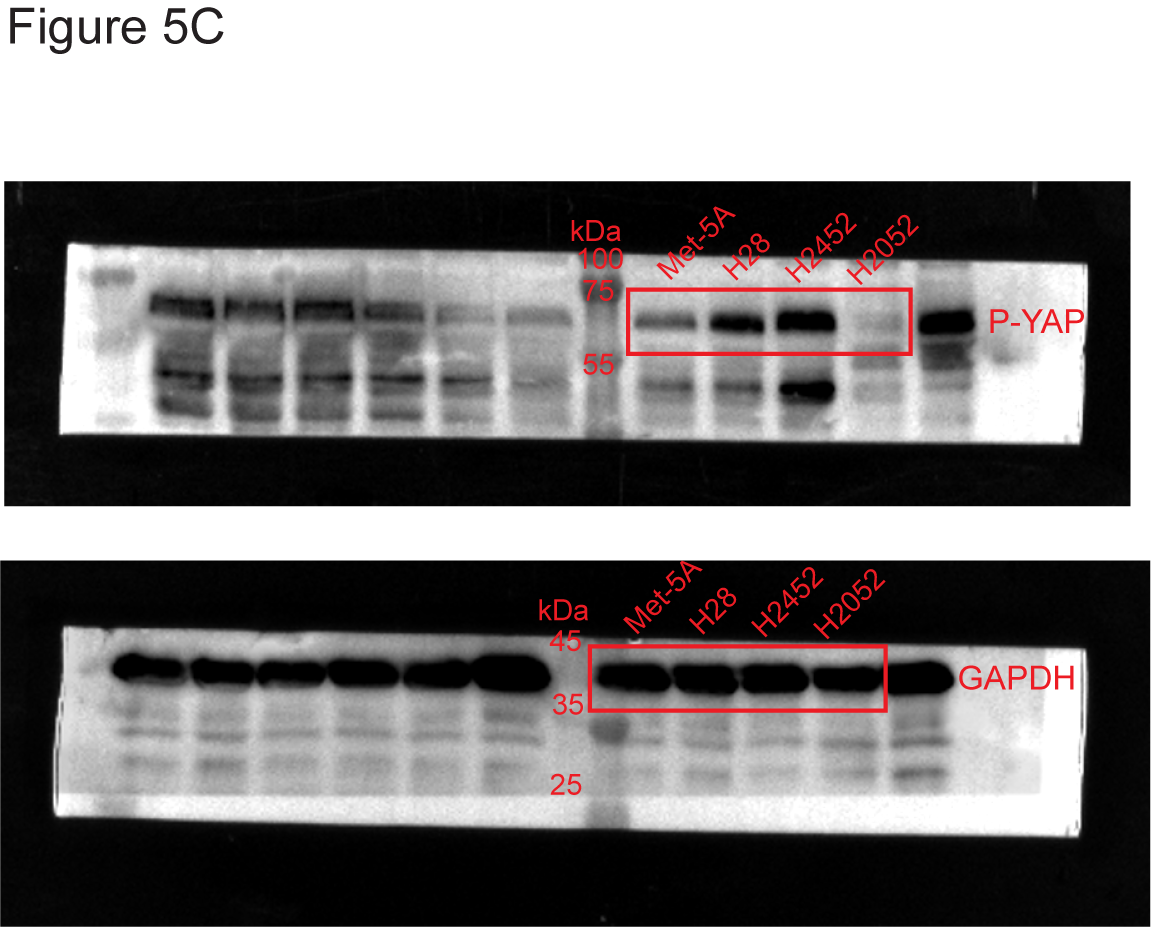

Supplement: Supplementary file 29 — Source data Fig. 5 [file 44321_2025_278_MOESM29_ESM.zip › Figure 5/5C/5C.tif]

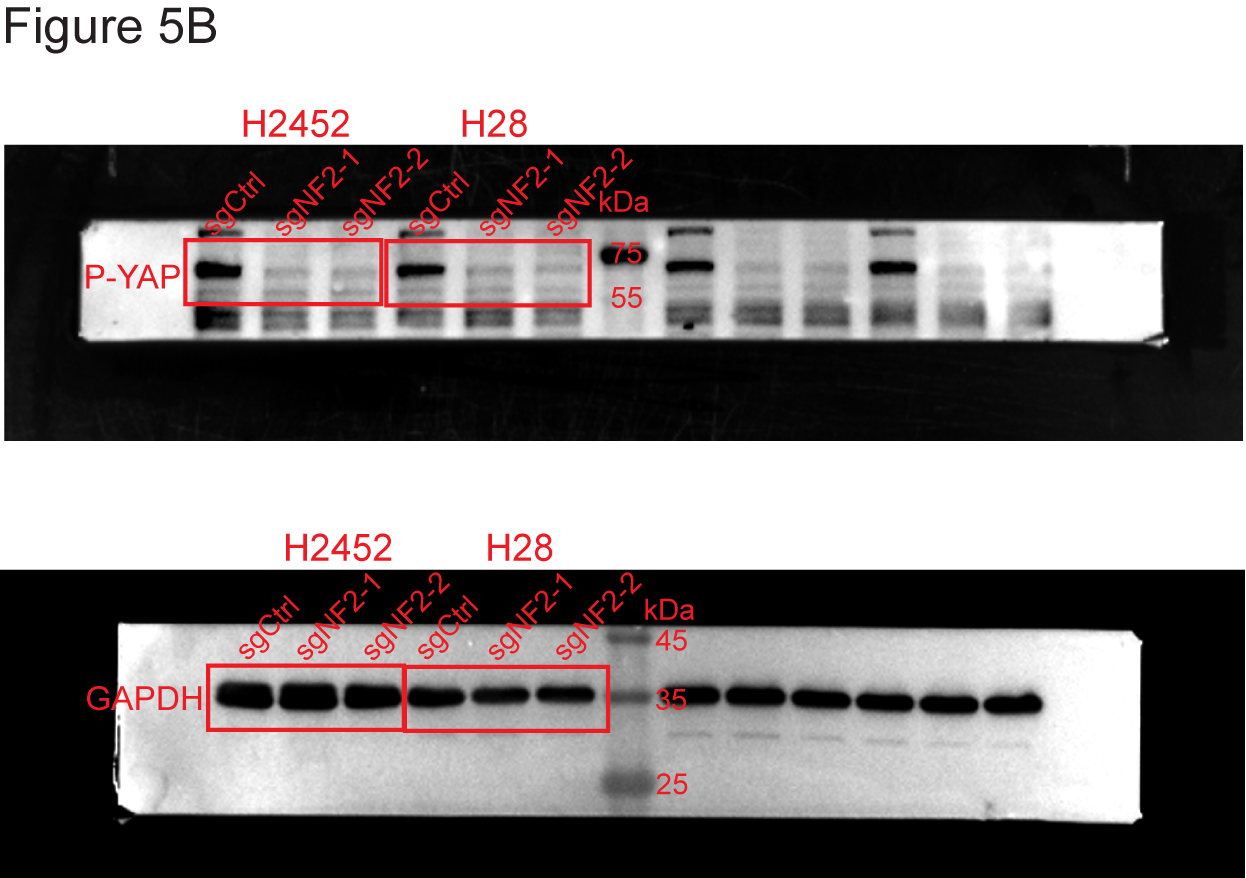

Supplement: Supplementary file 29 — Source data Fig. 5 [file 44321_2025_278_MOESM29_ESM.zip › Figure 5/5B/5B.tif]

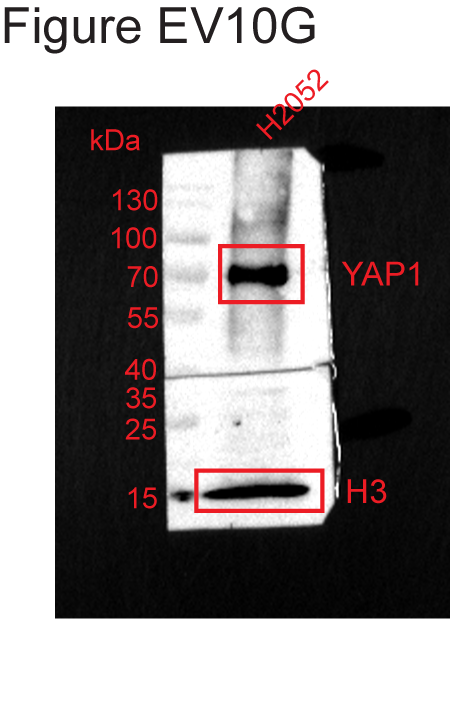

Supplement: Supplementary file 31 — Figure EV6 Source Data [file 44321_2025_278_MOESM31_ESM.zip › Figure EV6/G/EV10G.tif]

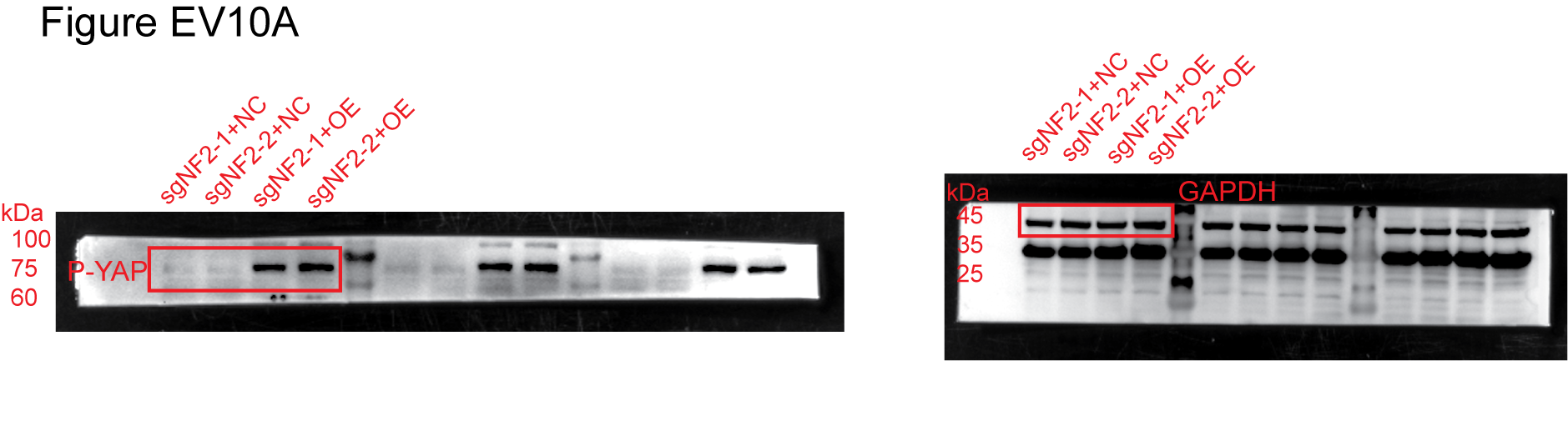

Supplement: Supplementary file 31 — Figure EV6 Source Data [file 44321_2025_278_MOESM31_ESM.zip › Figure EV6/A/EV10A.tif]

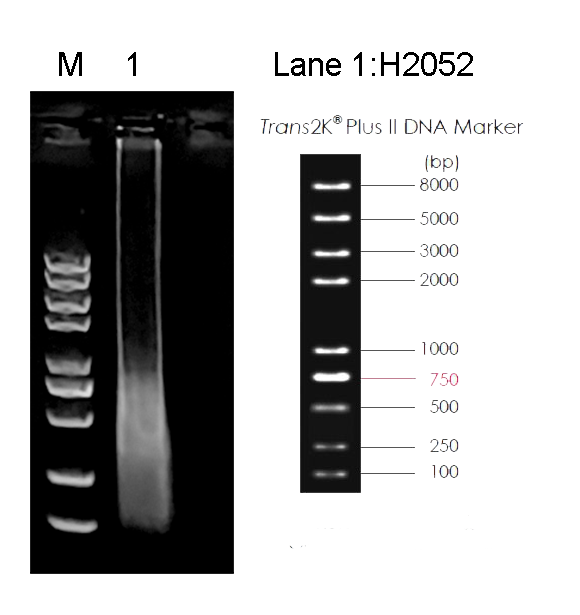

Supplement: Supplementary file 31 — Figure EV6 Source Data [file 44321_2025_278_MOESM31_ESM.zip › Figure EV6/H/EV10H.tif]

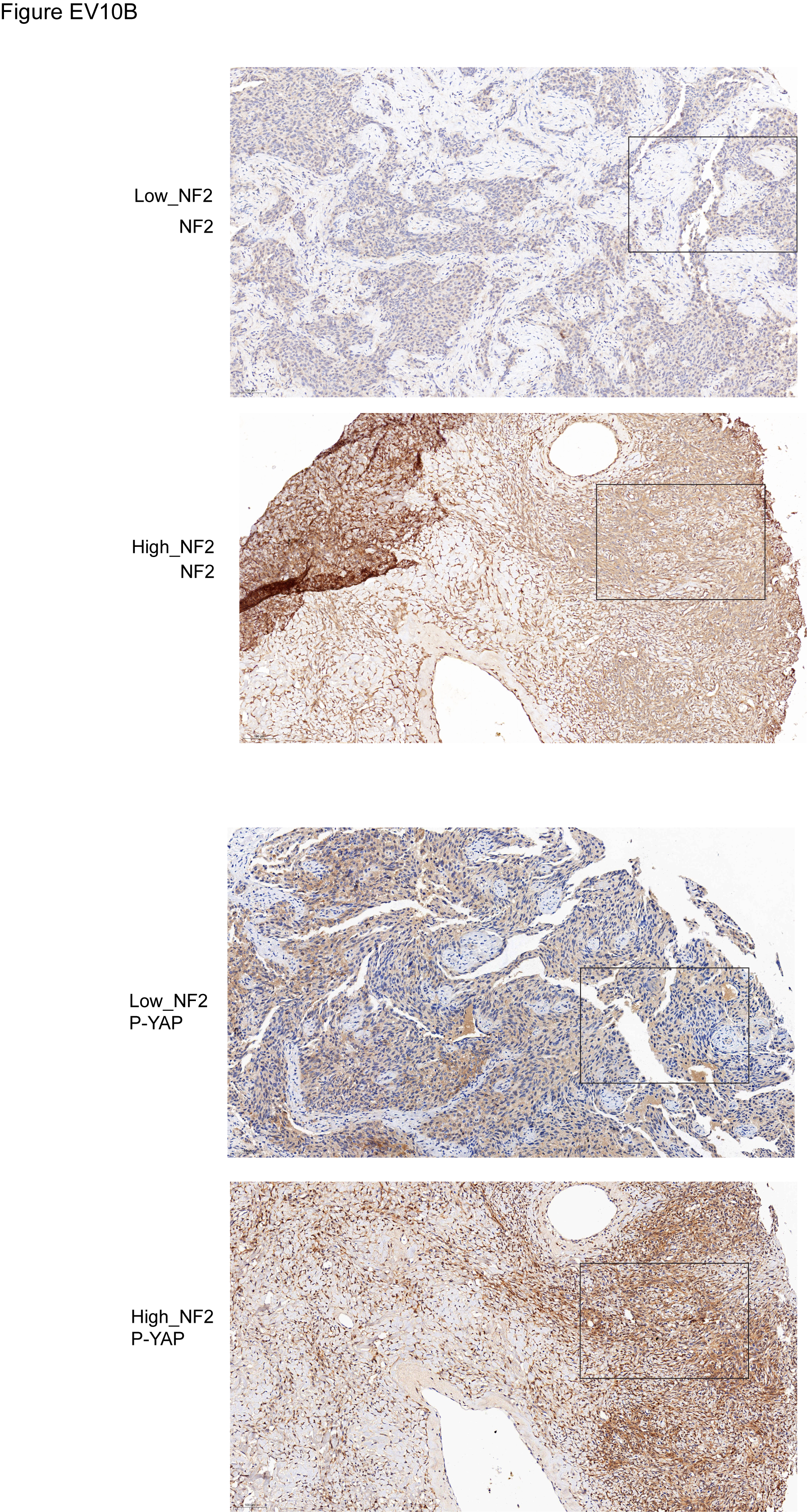

Supplement: Supplementary file 31 — Figure EV6 Source Data [file 44321_2025_278_MOESM31_ESM.zip › Figure EV6/B/EV10B.tif]
