# Supplementary material for: De novo pyrimidine synthesis is a collateral metabolic vulnerability in NF2-deficient mesothelioma
Source: EMBO Mol Med. 2025 Jul 24;17(9):2258–98. doi: 10.1038/s44321-025-00278-4 (PMC12423300; doi:10.1038/s44321-025-00278-4)
Supplement: Supplementary file 32 — Expanded View Figures [file 44321_2025_278_MOESM32_ESM.pdf]

Expanded View Figures

Figure EV1. ***NF2* deficiency promotes tumorigenesis in PM.**

(**A**) Immunoblots of the indicated proteins in H2452 (top) and H28 (bottom) PM cell lines transfected with a scrambled control (sgCtrl) or *NF2*-targeting sgRNAs (sg*NF2*-1, sg*NF2*-2). Representative images from three independent experiments are shown. Quantitative data can be found in Appendix Fig. S5A,B. (**B**) Cell viability assay of H2452 (left) and H28 (right) cells transfected with a scrambled control (sgCtrl) or *NF2*-targeting sgRNAs (sg*NF2*-1, sg*NF2*-2) at the indicated time points. The data are presented as the mean ± SD ($n = 3$). Two-way ANOVA with multiple comparisons was used for statistical analysis. (**C**, **D**) Clonogenic assay of H2452 (left) and H28 (right) cells transfected with a scrambled control (sgCtrl) or *NF2*-targeting sgRNAs (sg*NF2*-1, sg*NF2*-2). After being continually cultured for 14 days, viable cells were stained with crystal violet dye (**C**). (**D**) Quantification of the clonogenic assay results. The data are presented as the mean ± SD ($n = 3$). One-way ANOVA with multiple comparisons was used for statistical analysis. (**E**) Representative bioluminescence imaging (BLI) image showing tumor growth in athymic nu/nu mice across the indicated groups (H2452 sgCtrl and sg*NF2*-1) within an orthotopic mesothelioma model. (**F**) Quantification of BLI signals (photons/s) for the indicated groups (H2452 sgCtrl and sg*NF2*-1) in an orthotopic mesothelioma model using athymic nu/nu mice ($n = 9$ per group). The data are presented as the mean ± SEM. ns: not significant. Two-way ANOVA with multiple comparisons was used for statistical analysis. (**G**) Kaplan–Meier curves showing survival rates in the specified groups (H2452 sgCtrl and sg*NF2*-1) within an orthotopic mesothelioma model using athymic nu/nu mice ($n = 9$ per group). The *P* value was calculated by the log-rank test. (**H**) Kaplan–Meier curves showing overall survival (OS) in the TCGA cohort of mesothelioma patients ($n = 87$), stratified by the genetic status of *NF2*. The *P* value was calculated using the log-rank test in R. (**I**) Kaplan–Meier curves showing OS based on the protein levels of NF2 in the independent Bern cohort of mesothelioma patients ($n = 82$). Patients were stratified into high (in black) and low (in red) groups according to the optimal cut-off value of individual expression across the cohort using the surv_cutpoint function in the R "maxstat" package. The *P* value was calculated using the log-rank test in R.

© The Author(s)

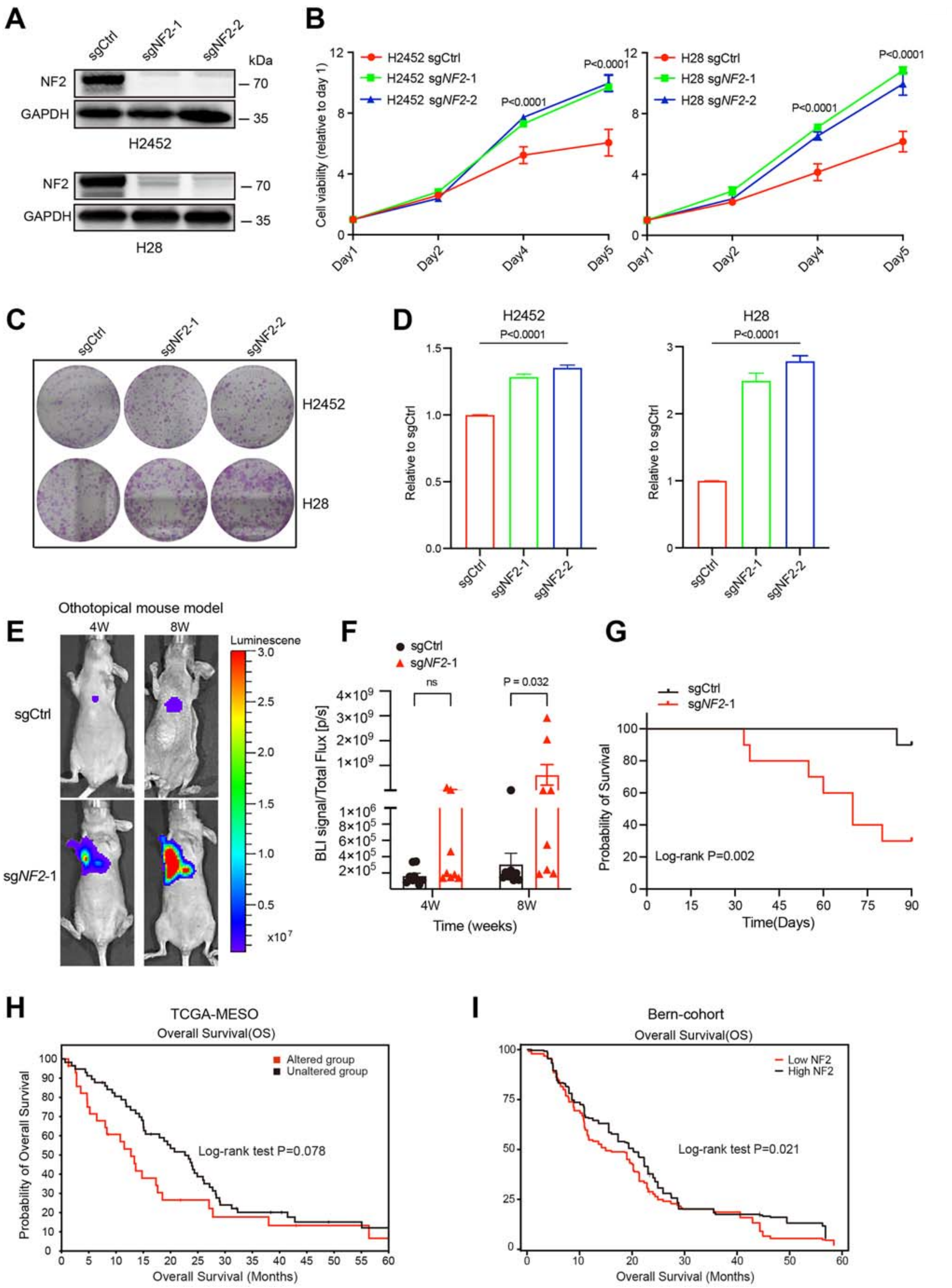
A
sgCtrl
sgNF2-1
sgNF2-2
kDa
NF2
GAPDH
70
35
H2452
H28
B
H2452 sgCtrl
H2452 sgNF2-1
H2452 sgNF2-2
H28 sgCtrl
H28 sgNF2-1
H28 sgNF2-2
Cell viability (relative to day 1)
P<0.0001
Day1
Day2
Day4
Day5
C
D
Relative to sgCtrl
E
Othotopical mouse model
4W
8W
Luminescene
x10^7
F
BLI signal/Total Flux [p/s]
ns
P = 0.032
Time (weeks)
G
Probability of Survival
Log-rank P=0.002
Time(Days)
H
TCGA-MESO
Overall Survival(OS)
Altered group
Unaltered group
Probability of Overall Survival
Log-rank test P=0.078
Overall Survival (Months)
I
Bern-cohort
Overall Survival(OS)
Low NF2
High NF2
Log-rank test P=0.021
Overall Survival (Months)

© The Author(s)

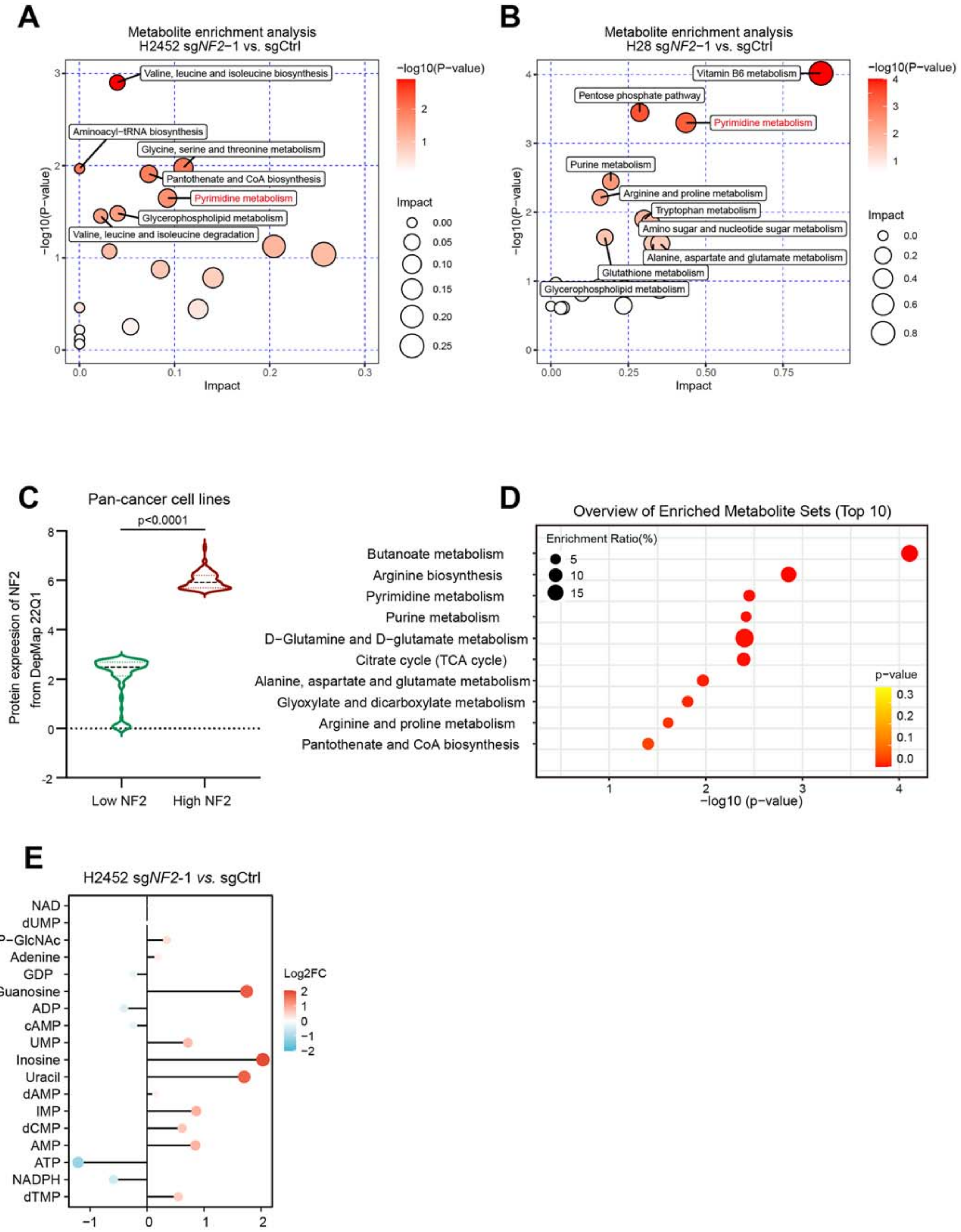
A
Metabolite enrichment analysis
H2452 sgNF2−1 vs. sgCtrl
Valine, leucine and isoleucine biosynthesis
Aminoacyl−tRNA biosynthesis
Glycine, serine and threonine metabolism
Pantothenate and CoA biosynthesis
Pyrimidine metabolism
Glycerophospholipid metabolism
Valine, leucine and isoleucine degradation
−log10(P−value)
Impact
B
Metabolite enrichment analysis
H28 sgNF2−1 vs. sgCtrl
Vitamin B6 metabolism
Pentose phosphate pathway
Pyrimidine metabolism
Purine metabolism
Arginine and proline metabolism
Tryptophan metabolism
Amino sugar and nucleotide sugar metabolism
Alanine, aspartate and glutamate metabolism
Glutathione metabolism
Glycerophospholipid metabolism
C
Pan-cancer cell lines
p<0.0001
Protein expreesion of NF2 from DepMap 22Q1
Low NF2
High NF2
D
Overview of Enriched Metabolite Sets (Top 10)
Enrichment Ratio(%)
Butanoate metabolism
Arginine biosynthesis
Pyrimidine metabolism
Purine metabolism
D−Glutamine and D−glutamate metabolism
Citrate cycle (TCA cycle)
Alanine, aspartate and glutamate metabolism
Glyoxylate and dicarboxylate metabolism
Arginine and proline metabolism
Pantothenate and CoA biosynthesis
p−value
−log10 (p−value)
E
H2452 sgNF2-1 vs. sgCtrl
NAD
dUMP
UDP−GlcNAc
Adenine
GDP
Guanosine
ADP
cAMP
UMP
Inosine
Uracil
dAMP
IMP
dCMP
AMP
ATP
NADPH
dTMP
Log2FC

 © The Author(s)

Figure EV2. *NF2* deficiency drives metabolic alterations in PM.

(**A**, **B**) Metabolite pathway enrichment analysis of differentially expressed metabolites with *P* value < 0.05 and a variable important in projection (VIP) > 1 in *NF2*-knockout (sg*NF2*-1) compared to control (sgCtrl) groups. The significantly altered metabolic pathways with *P* value < 0.05 are labeled in H2452 (**A**) and H28 (**B**). Pathway impact refers to the importance of altered metabolites in the respective metabolic pathway, as calculated by Metabo-Analyst. The analysis included six biological replicates for H2452, and three biological replicates for H28. Related to Fig. 1F. (**C**) The differential expression of NF2 between low-NF2 and high-NF2 groups across pan-cancer cell lines. The data were stratified according to the lowest and highest 100 NF2 protein expression levels in the ranking list, derived from the proteomic dataset available at the Cancer Dependency Map Data Portal. Protein expression is defined by the Z-score, which is calculated with reference to each protein as measured across the entire panel of cell lines. A two-tailed unpaired *t* test was used for comparisons. (**D**) Bubble diagram showing the top 10 enriched metabolite sets in the NF2-low group compared to the NF2-high group. (**E**) Lollipop chart depicting the differentially expressed metabolites (*P* value < 0.05) in the H2452 *sgNF2*-1 group compared to the sgCtrl group.

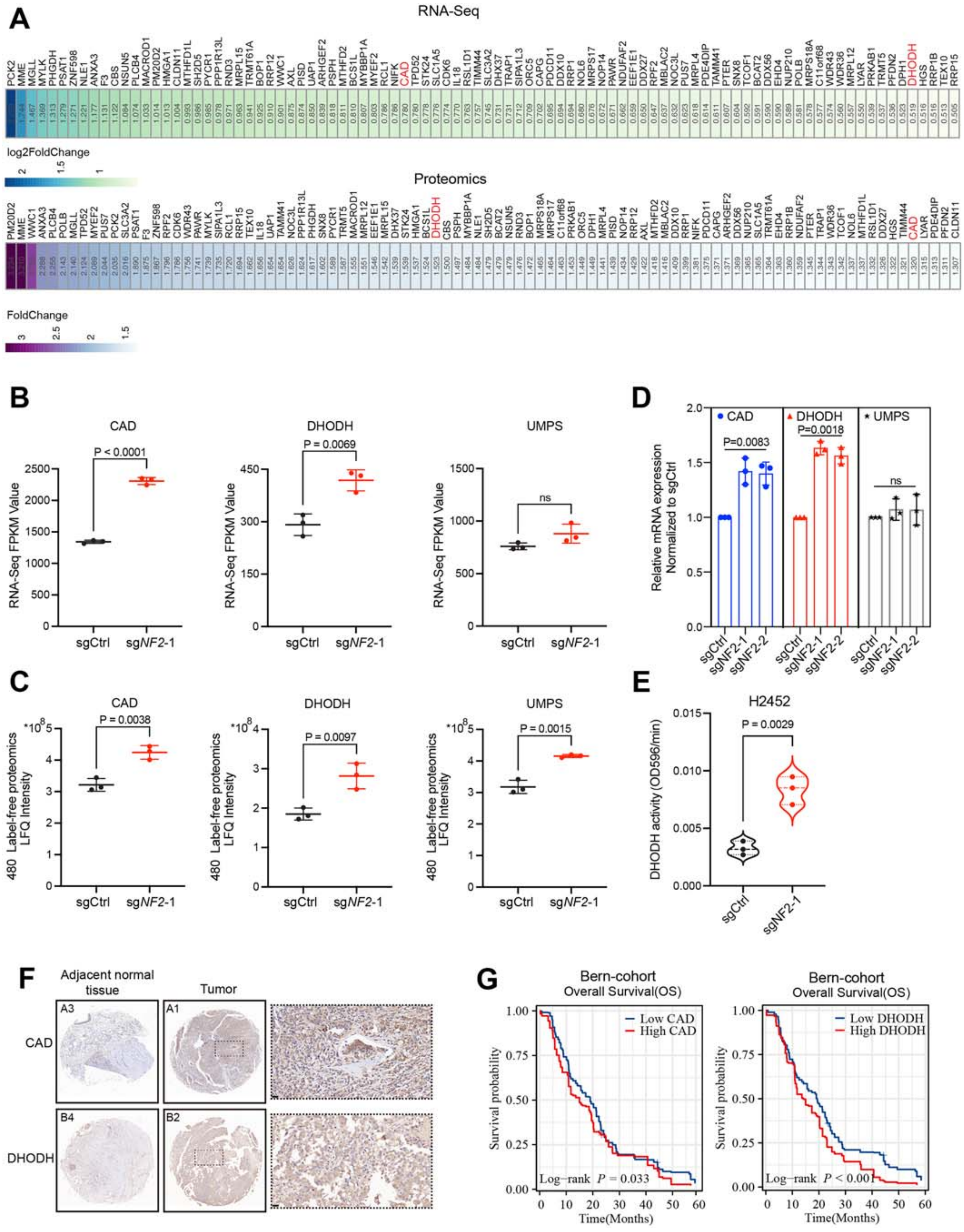
A
RNA-Seq
log2FoldChange
Proteomics
FoldChange
B
CAD
DHODH
UMPS
RNA-Seq FPKM Value
sgCtrl
sgNF2-1
C
480 Label-free proteomics LFQ Intensity
D
Relative mRNA expression Normalized to sgCtrl
sgNF2-2
E
H2452
DHODH activity (OD596/min)
F
Adjacent normal tissue
Tumor
G
Bern-cohort
Overall Survival(OS)
Low CAD
High CAD
Low DHODH
High DHODH
Survival probability
Time(Months)

Figure EV3. Loss of *NF2* elevates enzyme expression within the de novo pyrimidine synthesis pathway.

(**A**) Heatmap showing the 92 common candidates shared by both the differentially upregulated genes (P value < 0.05 and fold change>1.4) and proteins (P value < 0.05 and fold change>1.3) in the H2452 sg*NF2*-1 group compared to the sgCtrl group. Related to Fig. 2B. (**B**, **C**) The differences in FPKM values from RNA sequencing (**B**) and LFQ intensities from 480 label-free proteomes (**C**) of the indicated markers between the H2452 sg*NF2*-1 and sgCtrl groups. The data are presented as the mean ± SD ($n = 3$). A two-tailed unpaired t test was used for statistical analysis. (**D**) The mRNA expression of the indicated markers in H2452 PM cell lines transfected with a scrambled control (sgCtrl) or *NF2*-targeting sgRNAs (sg*NF2*-1, sg*NF2*-2). The data are presented as the mean ± SD ($n = 3$). One-way ANOVA with multiple comparisons was used for statistical analysis. (**E**) The enzymatic activity of DHODH in H2452 PM cell lines transfected with a scrambled control (sgCtrl) or *NF2*-targeting sgRNA (sg*NF2*-1). The data are presented as the mean ± SD ($n = 3$). A two-tailed unpaired t test was used for statistical analysis. (**F**) Representative images of immunohistochemistry (IHC) staining showing the expression of CAD and DHODH in paired normal tissue and tumor samples from the internal mesothelioma tissue microarray. Scale bar: 20 μm. (**G**) Kaplan–Meier curves showing OS based on the protein levels of CAD (left) and DHODH (right) in the independent Bern cohort of mesothelioma patients ($n = 82$). Patients were stratified into high (in black) and low (in red) according to the optimal cut-off value of individual expression across the cohort using the surv_cutpoint function in the R "maxstat" package. The P value was calculated using the log-rank test in R.

© The Author(s)

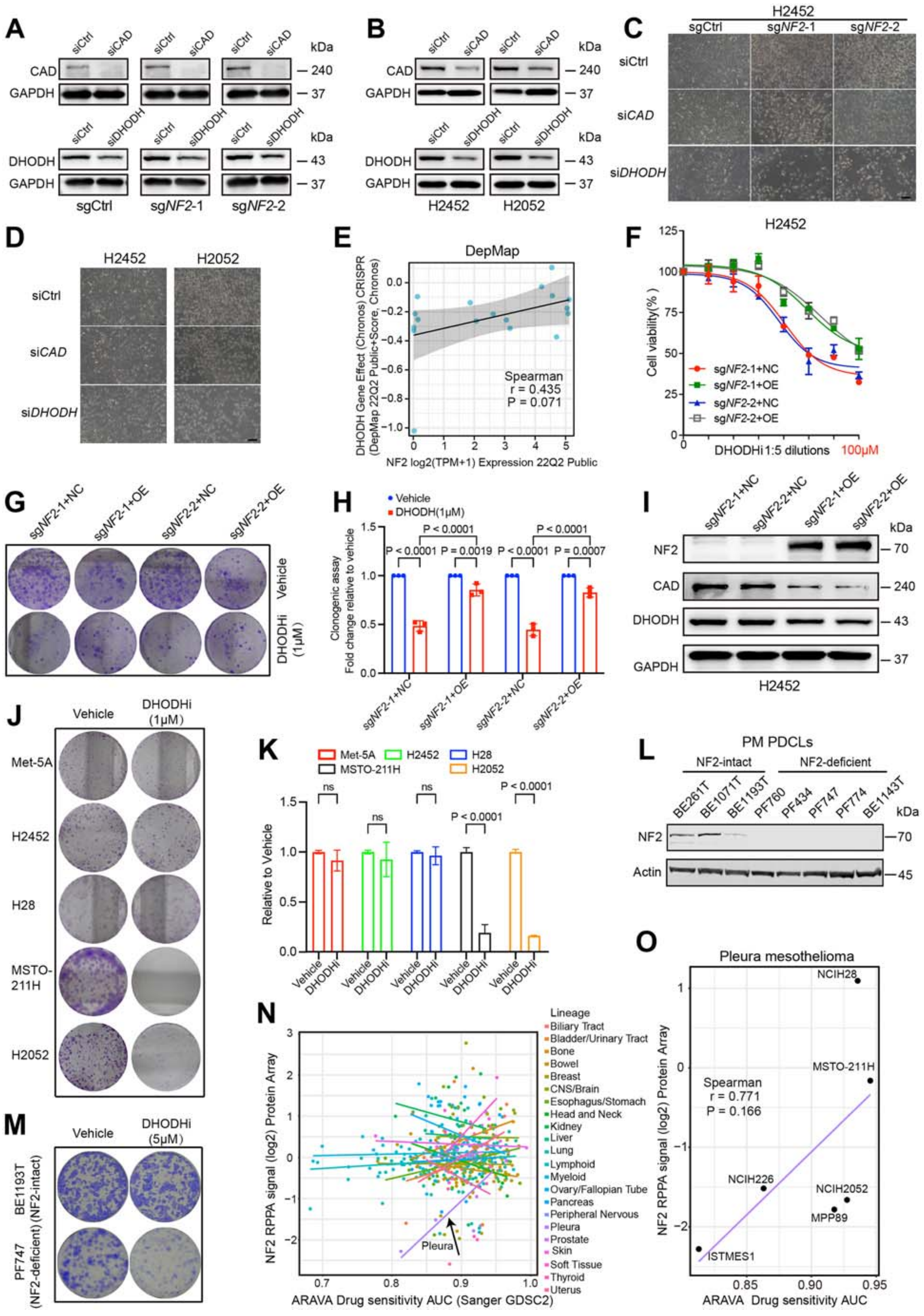
A
B
C
D
E
F
G
H
I
J
K
L
M
N
O
H2452
H2052
DepMap
Spearman
r = 0.435
P = 0.071
DHODH Gene Effect (Chronos) CRISPR (DepMap 22Q2 Public+Score, Chronos)
NF2 log2(TPM+1) Expression 22Q2 Public
Cell viability(%)
DHODHi 1:5 dilutions 100μM
Clonogenic assay Fold change relative to vehicle
Relative to Vehicle
PM PDCLs
NF2-intact
NF2-deficient
Pleura mesothelioma
Spearman
r = 0.771
P = 0.166
NF2 RPPA signal (log2) Protein Array
ARAVA Drug sensitivity AUC (Sanger GDSC2)
ARAVA Drug sensitivity AUC
Lineage
Pleura

Figure EV4. Targeting de novo pyrimidine synthesis selectively induces cell death in *NF2*-deficient PM cells.

(**A**) Immunoblots of the indicated proteins in H2452 sgCtrl, sg*NF2*-1, and sg*NF2*-2 PM cells after transfection with small interfering RNA (siRNA) targeting *CAD* or *DHODH* for 72 h. Representative images from three independent experiments are shown. Quantitative data can be found in Appendix Fig. S5H. (**B**) Immunoblots of the indicated proteins in H2452 (*NF2* wild-type) and H2052 (*NF2* mutant) cells after transfection with small interfering RNA (siRNA) targeting *CAD* or *DHODH* for 72 h. Representative images from three independent experiments are shown. Quantitative data can be found in Appendix Fig. S5I. (**C**) Representative images of the indicated cell populations after transfection with small interfering RNA (siRNA) targeting *CAD* or *DHODH* for 72 h. Scale bar: 100 μM. (**D**) Representative images of H2452 (*NF2* wild-type) and H2052 (*NF2* mutant) cells after transfection with small interfering RNA (siRNA) targeting *CAD* or *DHODH* for 72 h. Scale bar:100 μM. (**E**) Scatter plot showing the correlation between the transcript levels of NF2 and the genetic dependency score of DHODH in PM cell lines ($n = 18$). The data were obtained from the Dependency Map (22Q2). (**F**) H2452 sg*NF2*-1 and sg*NF2*-2 PM cells, transfected with a negative control (NC) or sgRNA-resistant NF2 cDNA (OE), were treated with increasing doses of the DHODH inhibitor Brequinar. Cell viability was measured 120 h post-treatment. The data are presented as the mean ± SD. Representative results from three independent experiments are shown. (**G**, **H**) Clonogenic assay of H2452 sg*NF2*-1 and sg*NF2*-2 PM cells transfected with a negative control (NC) or sgRNA-resistant NF2 cDNA (OE) and treated with vehicle or the DHODH inhibitor (1 μM) for 120 h. After a 14-day culture period, viable cells were stained with crystal violet. Representative images (**G**) and quantification (**H**) from three independent experiments are shown. The data are presented as the mean ± SD. Two-way ANOVA with multiple comparisons was used for statistical analysis. (**I**) Immunoblots of the indicated proteins in H2452 sg*NF2*-1 and sg*NF2*-2 PM cells transfected with an negative control (NC) or sgRNA-resistant NF2 cDNA (OE). Representative images from three independent experiments are shown. Quantitative data can be found in Appendix Fig. S5J. (**J, K**), Clonogenic assay of normal mesothelial cells (Met-5A) and PM cell lines (*NF2* wild-type: H2452, H28, MSTO-211H; *NF2* mutant: H2052) treated with vehicle or the indicated drugs for 96 h. After a 14-day culture period, the viable cells were stained with crystal violet. Representative images (**J**) and quantification (**K**) from three independent experiments are shown. The data are presented as the mean ± SD. Two-way ANOVA with multiple comparisons was used for statistical analysis. (**L**) Immunoblots of the indicated proteins in primary-derived cell lines (PDCLs) used for the cell viability assays shown in Appendix Fig. S5L. (**M**) Clonogenic assay of representative patient derived cell lines (PDCLs) BE1193T(NF2-intact) and PF747(NF2-deficient) treated with 1 μM DHODH inhibitor Brequinar for 96 h. After 14 days of cell growth, viable cells were fixed and stained with crystal violet dye. Representative images from three independent experiments are shown. (**N**, **O**) Scatter plot showing the correlation between NF2 protein levels and drug sensitivity to leflunomide (ARAVA; Sanger GDSC2) across a pan-cancer cohort of cell lines ($n = 1578$). Data from PM cell lines are presented separately in (**O**; $n = 6$). The data were generated from the DepMap project.

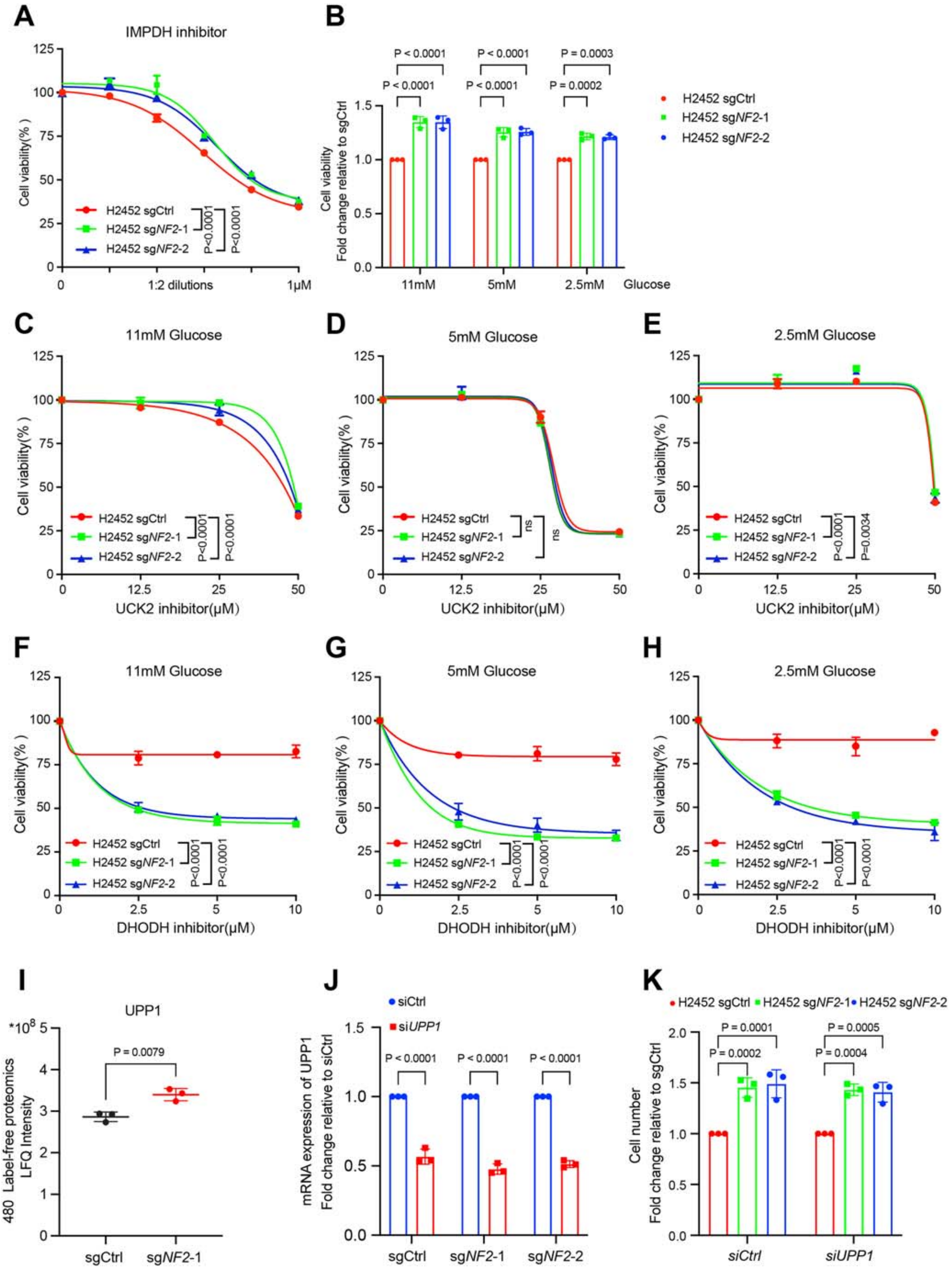
A
IMPDH inhibitor
Cell viability(%)
1:2 dilutions
1μM
H2452 sgCtrl
H2452 sgNF2-1
H2452 sgNF2-2
P<0.0001
P<0.0001
B
P < 0.0001
P < 0.0001
P = 0.0003
P < 0.0001
P < 0.0001
P = 0.0002
Cell viability
Fold change relative to sgCtrl
11mM
5mM
2.5mM
Glucose
H2452 sgCtrl
H2452 sgNF2-1
H2452 sgNF2-2
C
11mM Glucose
UCK2 inhibitor(μM)
D
5mM Glucose
ns
ns
E
2.5mM Glucose
P=0.0034
F
11mM Glucose
DHODH inhibitor(μM)
G
5mM Glucose
H
2.5mM Glucose
I
UPP1
*10^8
480 Label-free proteomics
LFQ Intensity
P = 0.0079
sgCtrl
sgNF2-1
J
siCtrl
siUPP1
mRNA expression of UPP1
Fold change relative to siCtrl
P < 0.0001
sgNF2-2
K
Cell number
Fold change relative to sgCtrl
P = 0.0001
P = 0.0002
P = 0.0005
P = 0.0004
siCtrl
siUPP1

 © The Author(s)

Figure EV5. ***NF2*****-deficient PM cells are independent of purine biosynthesis and pyrimidine salvage pathway.**

(**A**) Cell viability of H2452 *NF2* wild-type (sgCtrl) and *NF2* knockout (sg*NF2*-1, sg*NF2*-2) cells treated with various doses of the IMPDH inhibitor for 96 h. Representative data from three independent experiments are shown. The data are presented as the mean ± SD. Two-way ANOVA with multiple comparisons was used for statistical analysis. (**B**) Cell viability of H2452 *NF2* wild-type (sgCtrl) and *NF2* knockout (sg*NF2*-1, sg*NF2*-2) cells cultured with the indicated concentrations of glucose. The data are presented as the mean ± SD ($n = 3$). Two-way ANOVA with multiple comparisons was used for statistical analysis. (**C–E**) Cell viability of H2452 *NF2* wild-type (sgCtrl) and *NF2* knockout (sg*NF2*-1, sg*NF2*-2) cells treated with the indicated doses of the UCK2 inhibitor for 96 h at 10 mM (**C**), 5 mM (**D**) and 2.5 mM (**E**) glucose. Representative data from three independent experiments are shown. The data are presented as the mean ± SD. Two-way ANOVA with multiple comparisons was used for statistical analysis. (**F–H**) Cell viability of H2452 *NF2* wild-type (sgCtrl) and *NF2* knockout (sg*NF2*-1, sg*NF2*-2) cells treated with the indicated doses of the DHODH inhibitor for 96 h at 10 mM (**F**), 5 mM (**G**) and 2.5 mM (**H**) glucose. Representative data from three independent experiments were shown. The data are presented as the mean ± SD. Two-way ANOVA with multiple comparisons was used for statistical analysis. (**I**) The differences in LFQ intensities from 480 label-free proteomes of the indicated markers between the H2452 sg*NF2*-1 and sgCtrl groups. The data are presented as the mean ± SD ($n = 3$). A two-tailed unpaired *t* test was used for statistical analysis. (**J**, **K**) The mRNA expression of UPP1 (**J**) and cell viability (**K**) of the indicated cell populations transfected with small interfering RNA (siRNA) targeting the negative control or *UPP1* for 72 h. The data are presented as the mean ± SD ($n = 3$). Two-way ANOVA with multiple comparisons was used for statistical analysis.

© The Author(s)

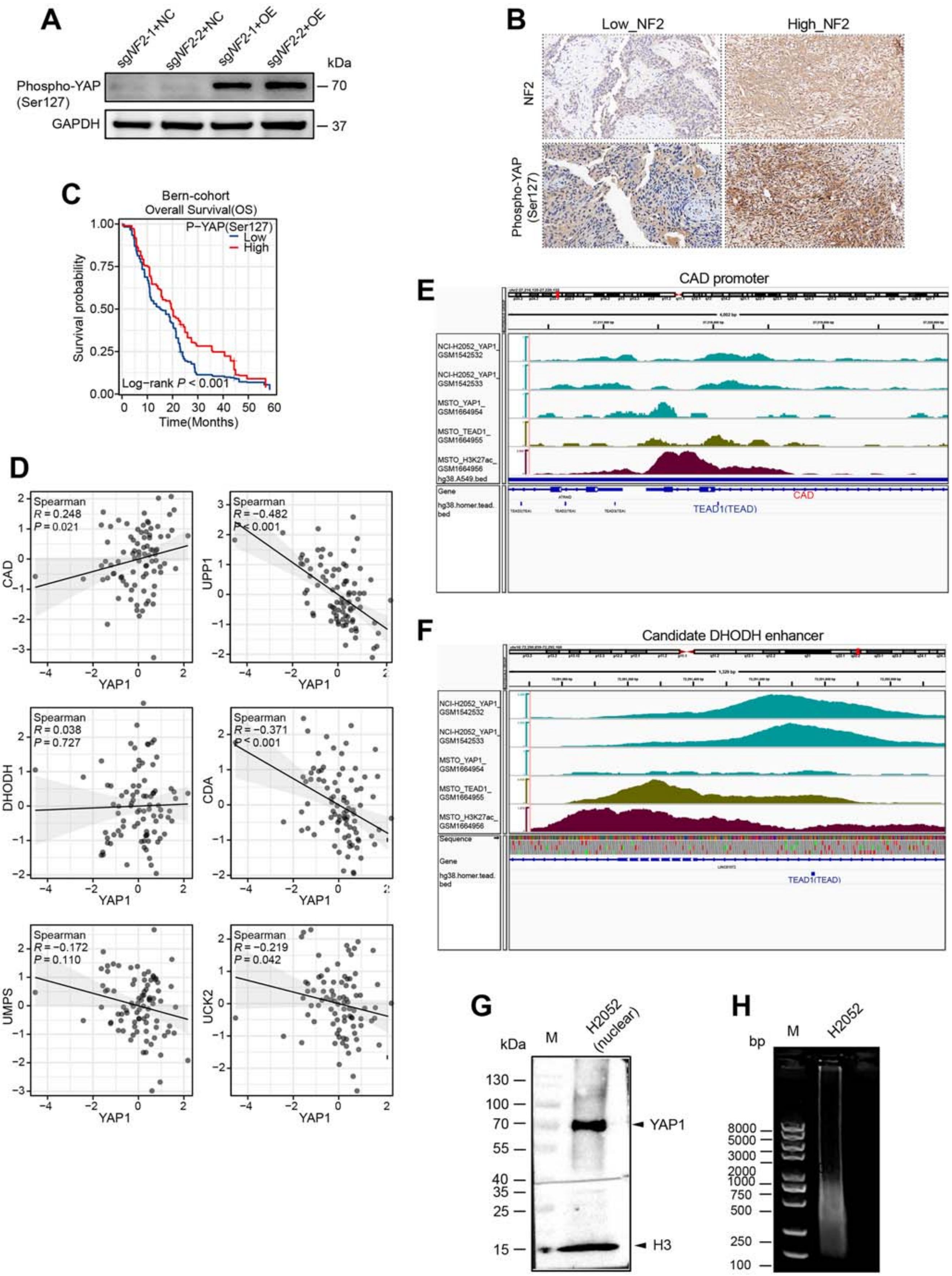
A
sgNF2-1+NC
sgNF2-2+NC
sgNF2-1+OE
sgNF2-2+OE
kDa
Phospho-YAP (Ser127)
— 70
GAPDH
— 37
B
Low_NF2
High_NF2
NF2
Phospho-YAP (Ser127)
C
Bern-cohort
Overall Survival(OS)
P-YAP(Ser127)
Low
High
Survival probability
Log-rank P < 0.001
Time(Months)
D
Spearman
R = 0.248
P = 0.021
CAD
YAP1
Spearman
R = −0.482
P < 0.001
UPP1
YAP1
Spearman
R = 0.038
P = 0.727
DHODH
YAP1
Spearman
R = −0.371
P < 0.001
CDA
YAP1
Spearman
R = −0.172
P = 0.110
UMPS
YAP1
Spearman
R = −0.219
P = 0.042
UCK2
YAP1
E
CAD promoter
NCI-H2052_YAP1_ GSM1542532
NCI-H2052_YAP1_ GSM1542533
MSTO_YAP1_ GSM1664954
MSTO_TEAD1_ GSM1664955
MSTO_H3K27ac_ GSM1664956
hg38.A549.bed
Gene
hg38.homer.tead. bed
CAD
TEAD1(TEAD)
F
Candidate DHODH enhancer
NCI-H2052_YAP1_ GSM1542532
NCI-H2052_YAP1_ GSM1542533
MSTO_YAP1_ GSM1664954
MSTO_TEAD1_ GSM1664955
MSTO_H3K27ac_ GSM1664956
Sequence
Gene
hg38.homer.tead. bed
TEAD1(TEAD)
G
kDa
M
H2052 (nuclear)
130
100
70
55
40
35
25
15
YAP1
H3
H
bp
M
H2052
8000
5000
3000
2000
1000
750
500
250
100

 © The Author(s)

Figure EV6. YAP enhances de novo pyrimidine synthesis through the transcriptional regulation of key enzymes.

(**A**) Immunoblots of the indicated proteins in H2452 sg*NF2*-1 and sg*NF2*-2 PM cells transfected with a negative control (NC) or sgRNA-resistant NF2 cDNA (OE). Representative images from three independent experiments are shown. Quantitative data can be found in Appendix Fig. S5K. (**B**) Representative IHC images of the indicated proteins in the low-NF2 and high-NF2 subgroups, stratified by the top and bottom quartiles of the H-score in our internal mesothelioma tissue microarray. The original overall magnification: ×200 (scale bar: 20 μm). (**C**) Kaplan–Meier curves showing OS based on the protein levels of P-YAP(Ser127) in the independent Bern cohort of mesothelioma patients ($n = 82$). Patients were stratified into high (in black) and low (in red) according to the optimal cut-off value of individual expression across the cohort using the surv_cutpoint function in the R "maxstat" package. The *P* value was calculated using the log-rank test in R. (**D**) Spearman correlation coefficients between the mRNA expression of YAP1 and other markers related to pyrimidine metabolism in the TCGA-MESO cohort ($n = 87$). (**E**, **F**) Representative YAP1/TEAD1-binding peak regions identified in the ChIP-seq datasets. The ChIP-seq data for YAP1/TEAD1 in the mesothelioma cell lines NCI-H2052 and MSTO-211H were retrieved from the Cistrome Data Browser (CistromeDB). The Y-axis is presented without normalization across the ChIP-seq datasets. (**G**) Immunoblots of nuclear YAP1 in the H2052 PM cell line. (**H**) Chromatin break agarose gel electrophoresis results for the H2052 PM cell line. Source data are available online for this figure.

© The Author(s)

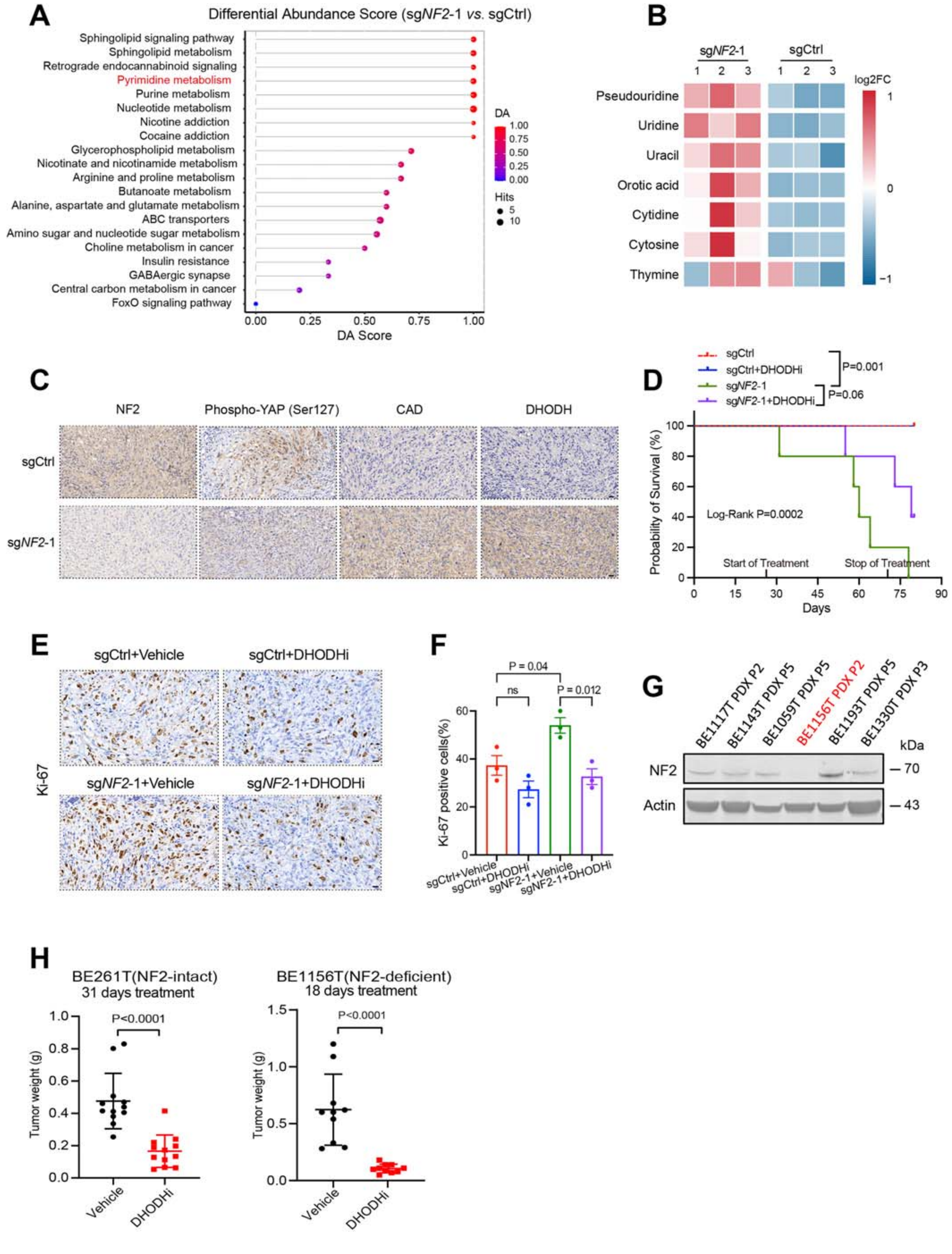
A
Differential Abundance Score (sgNF2-1 vs. sgCtrl)
Sphingolipid signaling pathway
Sphingolipid metabolism
Retrograde endocannabinoid signaling
Pyrimidine metabolism
Purine metabolism
Nucleotide metabolism
Nicotine addiction
Cocaine addiction
Glycerophospholipid metabolism
Nicotinate and nicotinamide metabolism
Arginine and proline metabolism
Butanoate metabolism
Alanine, aspartate and glutamate metabolism
ABC transporters
Amino sugar and nucleotide sugar metabolism
Choline metabolism in cancer
Insulin resistance
GABAergic synapse
Central carbon metabolism in cancer
FoxO signaling pathway
DA Score
DA
Hits
B
sgNF2-1
sgCtrl
Pseudouridine
Uridine
Uracil
Orotic acid
Cytidine
Cytosine
Thymine
log2FC
C
NF2
Phospho-YAP (Ser127)
CAD
DHODH
sgCtrl
sgNF2-1
D
sgCtrl
sgCtrl+DHODHi
sgNF2-1
sgNF2-1+DHODHi
P=0.001
P=0.06
Probability of Survival (%)
Log-Rank P=0.0002
Start of Treatment
Stop of Treatment
Days
E
sgCtrl+Vehicle
sgCtrl+DHODHi
sgNF2-1+Vehicle
sgNF2-1+DHODHi
Ki-67
F
P = 0.04
ns
P = 0.012
Ki-67 positive cells(%)
G
BE1117T PDX P2
BE1143T PDX P5
BE1059T PDX P5
BE1156T PDX P2
BE1193T PDX P5
BE1330T PDX P3
kDa
NF2
Actin
70
43
H
BE261T(NF2-intact)
31 days treatment
P<0.0001
Tumor weight (g)
Vehicle
DHODHi
BE1156T(NF2-deficient)
18 days treatment
P<0.0001

 © The Author(s)

Figure EV7. DHODH inhibition specifically reduces tumor growth in *NF2*-deficient PM mouse models.

(**A**) Differential abundance score (DA) reflecting the overall metabolic alterations between H2452 *sgNF2*-1 and sgCtrl-resected orthotopic tumors. A score of 1 indicates an upregulated expression pattern of metabolites identified in this pathway, and a score of -1 indicates a downregulated expression pattern. The length of the line segment represents the absolute value of the DA score, and the size of the dot at the end of the line segment represents the number of metabolites in the pathway. The depth of the color of the line segment and dot is proportional to the DA score: the darker the red color, the more inclined the overall expression of the pathway is to be upregulated; conversely the darker the blue color, the more inclined the overall expression is to be downregulated. (**B**) Heatmap showing the differentially expressed metabolites involved in pyrimidine metabolism (P value < 0.05 and VIP > 1) between H2452 *sgNF2*-1- and sgCtrl-resected orthotopic tumors ($n = 3$). (**C**) Representative IHC staining of the indicated proteins in resected H2452 sgCtrl or sg*NF2*-1 tumors. The original overall magnification is ×200 (scale bar: 20 μm). (**D**) Kaplan–Meier curves showing survival rates in the specified groups within an orthotopic mesothelioma model using NCG (NOD/ShiLtJGpt Prkdc^{em26Cd52}Il2rg^{em26Cd22}/Gpt) mice ($n = 5$ per group). The P value was calculated using the log-rank test. (**E**, **F**) IHC analysis of Ki-67 in resected H2452 tumors expressing sgCtrl or sg*NF2*-1 treated with or without the DHODH inhibitor (Brequinar; 30 mg/kg). The original overall magnification is ×200 (scale bar: 20 μm). Representative images (**E**) were captured and processed using Case Viewer software. The quantification of Ki-67-positive cells is shown in (**F**). The data are presented as the mean ± SD. Two-way ANOVA with multiple comparisons was used for statistical analysis. (**G**) Immunoblots of the indicated proteins in patient-derived xenograft (PDX) mesothelioma tumors. Cell lysates were obtained from snap-frozen PDX tumor tissues. (**H**) The tumor weights of PDX BE261T (left; $n = 6$ per group) and BE1156T (right; $n = 5$ per group) with and without the DHODH inhibitor treatment (Brequinar, 30 mg/kg, three times weekly). A two-tailed unpaired t test was used for statistical analysis.

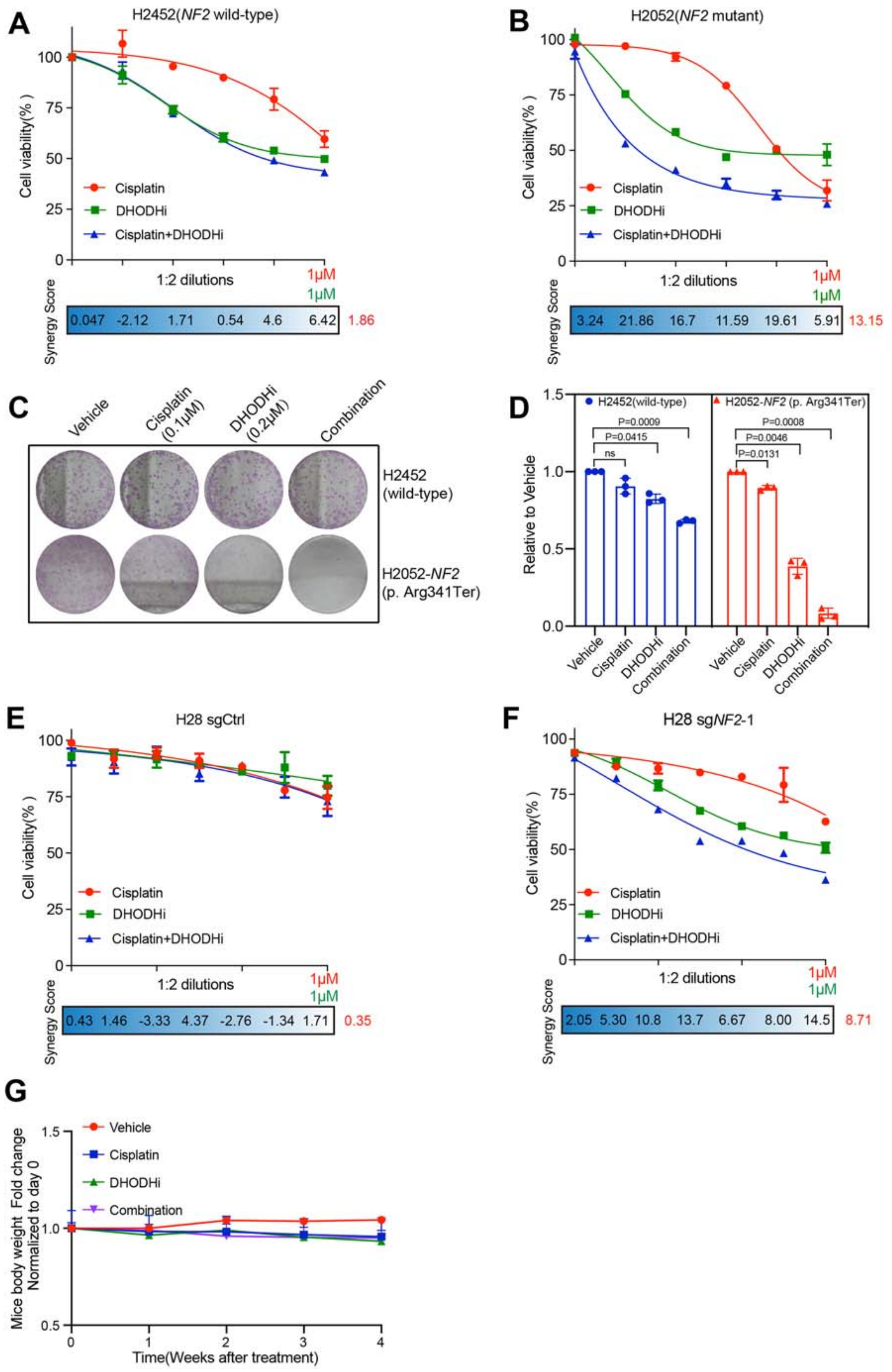
A
H2452(NF2 wild-type)
Cell viability(%)
Cisplatin
DHODHi
Cisplatin+DHODHi
1:2 dilutions
1μM
1μM
Synergy Score
0.047 -2.12 1.71 0.54 4.6 6.42 1.86
B
H2052(NF2 mutant)
Cell viability(%)
Cisplatin
DHODHi
Cisplatin+DHODHi
1:2 dilutions
1μM
1μM
Synergy Score
3.24 21.86 16.7 11.59 19.61 5.91 13.15
C
Vehicle
Cisplatin (0.1μM)
DHODHi (0.2μM)
Combination
H2452 (wild-type)
H2052-NF2 (p. Arg341Ter)
D
H2452(wild-type)
H2052-NF2 (p. Arg341Ter)
P=0.0009
P=0.0415
ns
P=0.0008
P=0.0046
P=0.0131
Relative to Vehicle
Vehicle
Cisplatin
DHODHi
Combination
E
H28 sgCtrl
Cell viability(%)
Cisplatin
DHODHi
Cisplatin+DHODHi
1:2 dilutions
1μM
1μM
Synergy Score
0.43 1.46 -3.33 4.37 -2.76 -1.34 1.71 0.35
F
H28 sgNF2-1
Cell viability(%)
Cisplatin
DHODHi
Cisplatin+DHODHi
1:2 dilutions
1μM
1μM
Synergy Score
2.05 5.30 10.8 13.7 6.67 8.00 14.5 8.71
G
Mice body weight Fold change Normalized to day 0
Vehicle
Cisplatin
DHODHi
Combination
Time(Weeks after treatment)

© The Author(s)

Figure EV8. DHODH inhibition exhibits a synergistic antitumour effect with cisplatin in PM.

(**A, B**) Cell viability of wild-type (H2452; **A**) and *NF2*-mutant (H2052; **B**) PM cell lines treated with the indicated doses of drugs for 96 h. Representative data from three independent experiments are shown. The data are presented as the mean ± SD. The highest single agent (HSA) synergy scores at the independent combination doses were calculated via SynergyFinder + . A synergistic score <−10 indicates that the interaction between two drugs is likely antagonistic; a score ranging from −10–10 indicates that the interaction is likely additive; and a score>10 indicates that the interaction is likely synergistic. (**C**, **D**) Clonogenic assay of H2452 (*NF2* wild-type) and H2052 (*NF2* mutant) PM cells treated with vehicle or the indicated drugs for 96 h. After a 14-day culture period, the viable cells were stained with crystal violet. Representative images (**C**) and quantification (**D**) from three independent experiments are shown. The data are presented as the mean ± SD. Two-way ANOVA with multiple comparisons was used for statistical analysis. (**E**, **F**) Cell viability of wild-type (H28 sgCtrl; **E**) and *NF2*-deficient PM cell lines (H28 sg*NF2*-1; **F**) treated with the indicated doses of drugs for 96 h ($n = 3$). The data are presented as the mean ± SD. Representative results from three independent experiments are shown. (**G**) The body weights of mice during the indicated experiment. The data are presented as the mean ± SEM ($n = 3$ or 4 per group).

© The Author(s)
