# Supplementary figures and images for: Correction to: De novo pyrimidine synthesis is a collateral metabolic vulnerability in NF2-deficient mesothelioma
Source: EMBO Mol Med. 2026 Jan 16;18(2):851–4. doi: 10.1038/s44321-025-00366-5 (PMC12905169; doi:10.1038/s44321-025-00366-5)

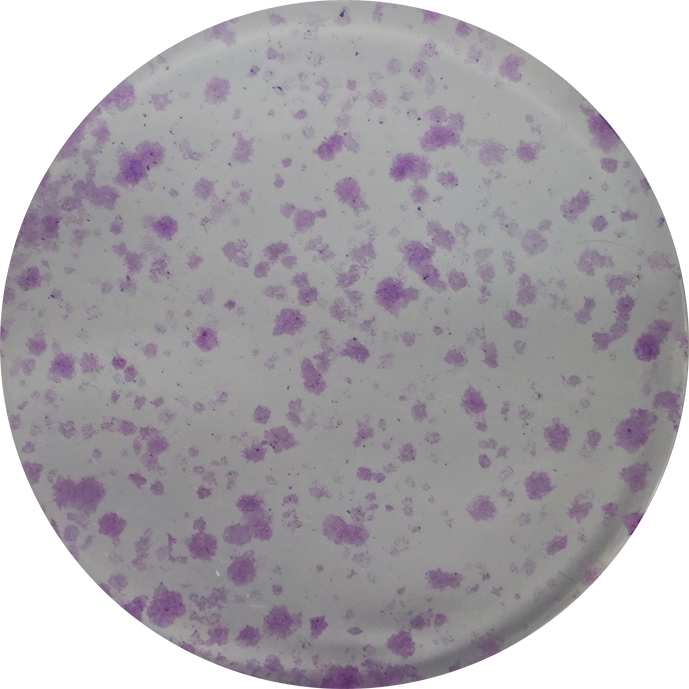

Supplement: Supplementary file 1 — Figure EV1C Source Data [file 44321_2025_366_MOESM1_ESM.zip › Figure EV1C Source Data/H2452 sgNF2-2_Raw.tif]

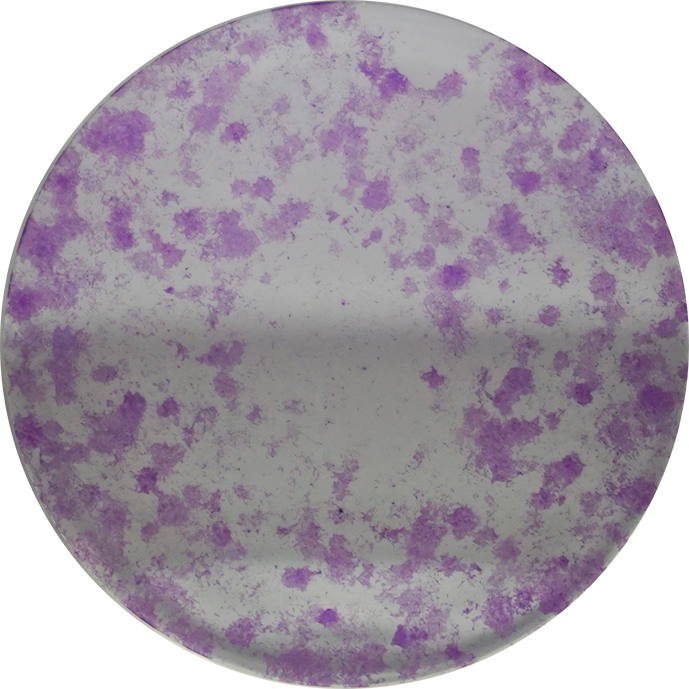

Supplement: Supplementary file 1 — Figure EV1C Source Data [file 44321_2025_366_MOESM1_ESM.zip › Figure EV1C Source Data/H28 sgNF2-1_Raw.tif]

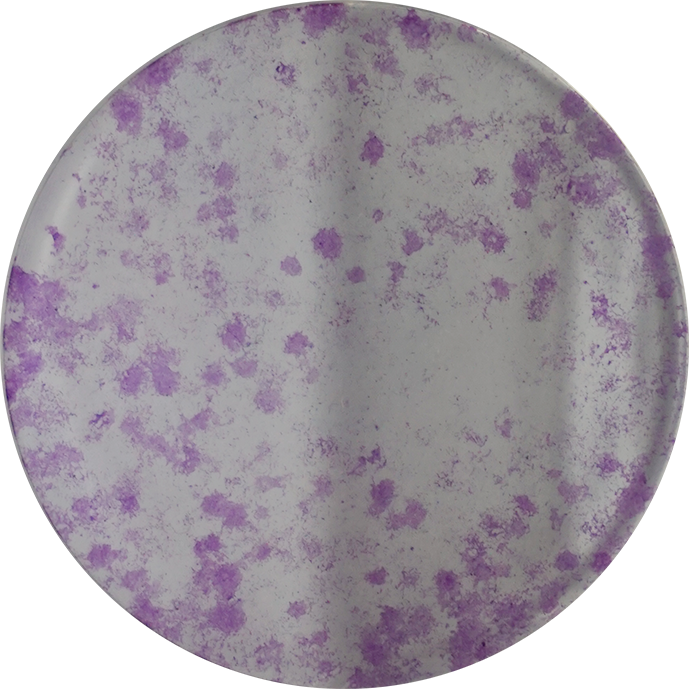

Supplement: Supplementary file 1 — Figure EV1C Source Data [file 44321_2025_366_MOESM1_ESM.zip › Figure EV1C Source Data/H28 sgCtrl_Raw.tif]

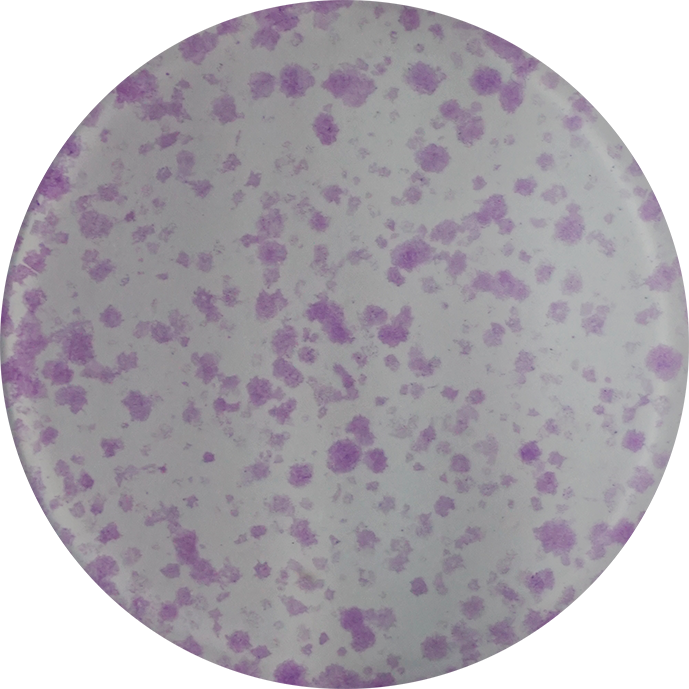

Supplement: Supplementary file 1 — Figure EV1C Source Data [file 44321_2025_366_MOESM1_ESM.zip › Figure EV1C Source Data/H2452 sgNF2-1_Raw.tif]

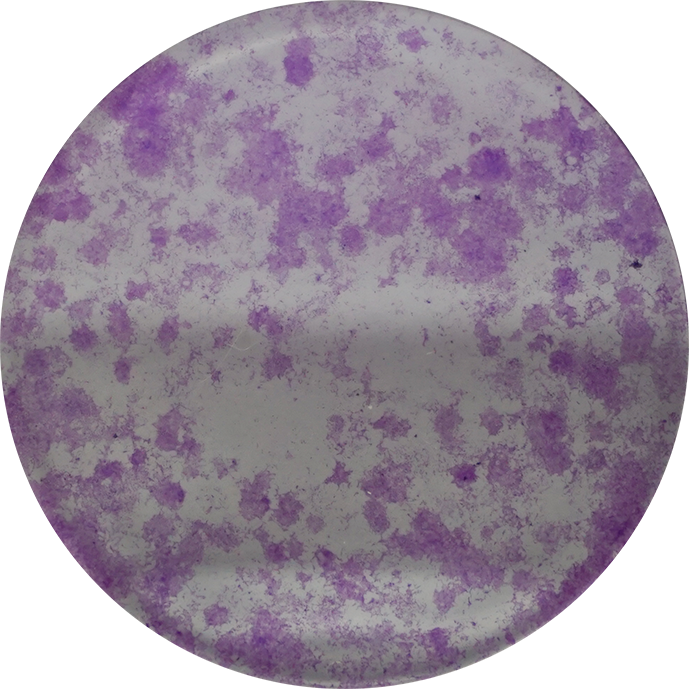

Supplement: Supplementary file 1 — Figure EV1C Source Data [file 44321_2025_366_MOESM1_ESM.zip › Figure EV1C Source Data/H28 sgNF2-2_Raw.tif]

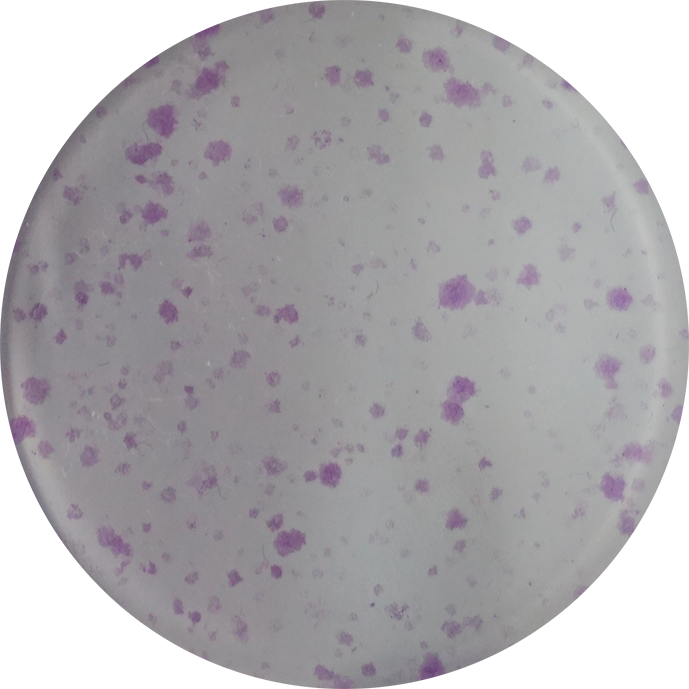

Supplement: Supplementary file 1 — Figure EV1C Source Data [file 44321_2025_366_MOESM1_ESM.zip › Figure EV1C Source Data/H2452 sgCtrl_Raw.tif]
